# Nucleus pulposus cells regulate macrophages in degenerated intervertebral discs via the integrated stress response-mediated CCL2/7-CCR2 signaling pathway

**DOI:** 10.1038/s12276-024-01168-4

**Published:** 2024-02-05

**Authors:** Shuo Tian, Xuanzuo Chen, Wei Wu, Hui Lin, Xiangcheng Qing, Sheng Liu, BaiChuan Wang, Yan Xiao, Zengwu Shao, Yizhong Peng

**Affiliations:** 1 Department of Orthopedics, Union Hospital, Tongji Medical College, Huazhong University of Science and Technology, Wuhan, 430022 China; 2 Department of Orthopedics, Peking University Third Hospital, Beijing, China; 3 Department of Radiology, Union Hospital, Tongji Medical College, Huazhong University of Science and Technology, Wuhan, 430022 China

**Keywords:** Chemokines, Mechanisms of disease, Cartilage, Chronic inflammation

## Abstract

Lower back pain (LBP), which is a primary cause of disability, is largely attributed to intervertebral disc degeneration (IDD). Macrophages (MΦs) in degenerated intervertebral discs (IVDs) form a chronic inflammatory microenvironment, but how MΦs are recruited to degenerative segments and transform into a proinflammatory phenotype remains unclear. We evaluated chemokine expression in degenerated nucleus pulposus cells (NPCs) to clarify the role of NPCs in the establishment of an inflammatory microenvironment in IDD and explored the mechanisms. We found that the production of C-C motif chemokine ligand 2 (CCL2) and C-C motif chemokine ligand 7 (CCL7) was significantly increased in NPCs under inflammatory conditions, and blocking CCL2/7 and their receptor, C-C chemokine receptor type 2(CCR2), inhibited the inductive effects of NPCs on MΦ infiltration and proinflammatory polarization. Moreover, activation of the integrated stress response (ISR) was obvious in IDD, and ISR inhibition reduced the production of CCL2/7 in NPCs. Further investigation revealed that activating Transcription Factor 3 (ATF3) responded to ISR activation, and ChIP-qPCR verified the DNA-binding activity of ATF3 on CCL2/7 promoters. In addition, we found that Toll-like receptor 4 (TLR4) inhibition modulated ISR activation, and TLR4 regulated the accumulation of mitochondrial reactive oxygen species (mtROS) and double-stranded RNA (dsRNA). Downregulating the level of mtROS reduced the amount of dsRNA and ISR activation. Deactivating the ISR or blocking CCL2/7 release alleviated inflammation and the progression of IDD in vivo. Moreover, MΦ infiltration and IDD were inhibited in CCR2-knockout mice. In conclusion, this study highlights the critical role of TLR4/mtROS/dsRNA axis-mediated ISR activation in the production of CCL2/7 and the progression of IDD, which provides promising therapeutic strategies for discogenic LBP.

## Inflammatory Responses: The Hidden Culprit Behind Intervertebral Disc Degeneration

Low back pain, a main reason for disability, is often due to intervertebral disc degeneration (IDD - the wear and tear of the discs between the vertebrae of the spine). This degeneration, accounting for about 40% of chronic low back pain instances, results in the damage of spinal structure and function. In this research, scientists explored the role of inflammation in IDD, focusing on the role of macrophages. They found that macrophages with different polarized phenotypes (observable traits) existed in degenerative intervertebral disc tissue. The scientists also found that the polarization of these macrophages turned proinflammatory, which intensified the inflammatory cascade reaction (series of biochemical events that lead to inflammation), worsening inflammation and degeneration of the intervertebral disc. This research offers new understanding into the role of inflammation in IDD and potential intervention targets. This summary was initially drafted using artificial intelligence, then revised and fact-checked by the author.

## Introduction

Low back pain (LBP), which is a leading cause of disability and symptomatic intervertebral disc (IVD), imposes heavy economic and social burdens^1^. Intervertebral disc degeneration (IDD) contributes to approximately 40% of chronic LBP cases^2^. IDD leads to the destruction of the structure and mobility of the spine, ultimately leading to pain and loss of function^3^. Recent evidence shows that inflammation plays an important role in IDD^4^. A degenerated IVD forms an inflammatory microenvironment that contains a variety of cytokines, including C-C motif chemokine ligand 2 (CCL2) and C-C motif chemokine ligand 7 (CCL7)^5,6^. Due to the integrity of the annulus fibrosus (AF), nucleus pulposus (NP) tissue is physically isolated from the immune system and is an immune privileged site under physiological conditions^7^, and many immune cells are attracted by these inflammatory cytokines and infiltrate through AF fissures in degenerative conditions^8,9^; macrophages (MΦs) with different polarized phenotypes have been confirmed to exist in degenerative IVD tissue^10^. With the aggravation of IDD, the polarization of MΦs becomes proinflammatory, which further amplifies the inflammatory cascade reaction, thereby exacerbating inflammation and degeneration of the IVD^5,10^. Although MΦs play a critical role in the progression of IDD, the mechanism of MΦ recruitment and polarization in IVDs remains unclear^11^.

Cell stress is a general term for a series of reactions when cells encounter various internal and external environmental stimuli, including hunger, temperature change, hypoxia, and oxidative stress^12^. The integrated stress response (ISR), which is a series of evolutionarily conserved intracellular signaling networks, responds to various stimuli and sequentially modifies the phosphorylation of eukaryotic initiation factor 2α subunit (eIF2α), ultimately triggering changes in global protein synthesis to quickly affect protein levels^13^. Specifically, activation of the ISR leads to increased eIF2α phosphorylation at the Ser-51 site and inhibits protein translation to enhance the adaptive capacity to support cell survival. It can also increase the expression of stress proteins such as activating transcription factor 4 (ATF4)^14^. The ISR enables cells to resist stress and restore tissue homeostasis^15^. However, when stress continues, ISR enters the chronic phase and is a primary pathophysiological factor for many disease conditions, including peripheral neuropathy and ischemic heart damage^16,17^. Four kinases, including the serine/threonine-protein kinase GCN2, protein kinase R (PKR), PKR-like endoplasmic reticulum kinase (PERK), and heme-regulated inhibitor (HRI), mediate activation of the ISR, and PERK-mediated endoplasmic reticulum stress has been reported to participate in the development and progression of IDD^18^. To our knowledge, few studies have systematically explored the mechanisms of ISR activation in IDD^19^. Investigating the role of the ISR in regulating IVD inflammation will further clarify the mechanism of IDD and provide novel therapeutic targets.

In this study, we aimed to explore the effects of the ISR on the establishment of IVD inflammation and found that nucleus pulposus cells (NPCs) recruited MΦs to the IVD region through the CCL2/7-C-C chemokine receptor type 2 (CCR2) pathway and promoted M1 polarization, which depended on the PKR-mediated ISR and activating transcription factor 3 (ATF3)-mediated CCL2/7 transcription in NPCs. We further identified that mitochondrial reactive oxygen species (mtROS) accumulation and subsequent generation of endogenous dsRNA caused PKR-mediated ISR activation. Therefore, our results demonstrated that activation of the ISR in NPCs was involved in MΦ infiltration and proinflammatory polarization during IDD, providing novel targets for IDD intervention.

## Materials and methods

### Collection of human NP samples

The present study was approved by the Medical Ethics Committee of Tongji Medical College at Huazhong University of Science and Technology (HUST). All patients were aware of the experimental protocols and their rights. NP tissues were collected from patients with lumbar idiopathic scoliosis, spinal stenosis, and lumbar disc herniation during routine lumbar disc surgery after the patients provided written informed consent. The severity of IDD was evaluated according to the Pfirrmann grading system, and samples with Pfirrmann grades less than III were regarded as controls^20^, while the others were considered degenerative.

### Cell culture

The extraction of NPCs was performed as previously reported^21–23^. To isolate rat NPCs, 8-week-old male SD rats weighing 200–230 g were collected. NP tissue was extracted from rat caudal IVD tissue and cut on an ultraclean platform. To isolate human NPCs, surgical samples were partially obtained from the previously described control group, washed and cut on an ultraclean platform. To isolate mouse NPCs, 6-week-old male mice were sacrificed, and a straight vertical incision was made from the caudal end along the midline over the spine toward the rostral end to dissect the spine. Then, the soft tissue covering the side of the spine was removed to expose the discs, and mouse NP tissue was collected using a feather surgical blade.

The obtained NP tissues were digested with 0.25% type II collagenase (Gibco, USA) and then cultured in DMEM/F12 medium (Gibco, USA) containing 20% fetal bovine serum (ScienCell, USA). The medium was changed every 3 days. When the NPCs grew to 90% confluence, 0.25% trypsin (Gibco, USA) was used for passaging, and the cells were cultured in DMEM/F12 medium (Gibco, USA) containing 10% fetal bovine serum (ScienCell, USA). Second-generation cells were used for the experiment. All cells were cultured in a 37 °C incubator containing 5% CO_2_.

For rat/mouse primary MΦs, the femur was removed under aseptic conditions; the bone marrow was flushed out with a syringe and then centrifuged and resuspended after being treated with red blood cell lysis buffer (Beyotime, China). Rat MΦs were cultured with 10% RPMI-1640 (Gibco, USA) containing 25 ng/ml rat M-CSF (AF-400-28-10, PeproTech, USA). Mouse MΦs were cultured with mouse M-CSF (AF-315-02, PeproTech, USA) and high glucose DMEM (Gibco, USA). The culture medium was changed every 3 days.

### Cell treatments

LPS was used to establish the degenerative model in vitro to simulate the inflammatory environment in which DAMPs or PAMPs exists^24–26^. Specifically, NPCs were pretreated with drugs (ISRIB, 1 µM; bindarit, 300 µM; PKR-IN-C16, 1 µM; GCN2iB, 2 µM; GSK2606414, 1 µM; TLR4-IN-C34, 10 µM; MitoTempo, 20 µM; IMT1B, 200 nM) for 1 h before LPS (1 µg/ml, Sigma, USA) was added, and the cells were cultured for another 6 h before further analysis. To obtain degenerative supernatant, NPCs were treated with LPS (1 µg/ml) for 6 h and washed with PBS 3 times. Then, the cells were cultured with serum-free RPMI 1640 medium (Gibco, USA) for another 24 h. Finally, the supernatant was collected and denoted as degenerative supernatant.

The supernatant from NPCs treated with serum-free RPMI 1640 medium for 24 h was collected as a control. The obtained supernatant was used to treat MΦs or for ELISA experiments. MΦs were cultured with supernatant derived from NPCs with or without RS102895 (2 µM) treatment for 24 h before further evaluation. ISRIB, bindarit, RS102895, PKR-IN-C16, GCN2iB, GSK2606414, TLR4-IN-C34, and MitoTempo were purchased from Selleck (Selleck Chemicals LLC, Texas, USA). IMT1B was purchased from MCE (MedChemExpress, New Jersey, USA).

### Total RNA extraction, RT-PCR and qPCR

RNA was extracted from cells cultured in six-well plates after the indicated treatments. RNA-easy isolation reagent (Vazyme, China) was used for RNA extraction. The extracted RNA was reverse transcribed with a HiScript III kit (R323, Vazyme, China). The obtained cDNA was subjected to qPCR using SYBR Green Mix (Q111, Vazyme, China), relative gene expression was calculated using the 2^-ΔΔCq method, and GAPDH was used as the internal reference. All processes followed the manufacturer’s standard operating procedures. The qPCR primers were designed using NCBI Primer-Blast and are listed in the supplementary materials (Supplementary Table 1).

### Mitochondrial ROS analysis

Treated NPCs were washed with PBS 3 times and stained with the MitoSOX probe (Introvigen, USA) according to the protocol provided by the manufacturer. After staining, the signal was visualized by a fluorescence microscope (Olympus, Tokyo, Japan). For FCM analysis, 0.25% trypsin was used to digest the treated cells in flow tubes containing 3 × 10^4^ cells/tube. After being stained with the MitoSOX probe, the cells were evaluated by a CytoFlex flow cytometer (Beckman, USA) and analyzed by FlowJo software.

### Transwell assay

MΦ migration was detected in a Transwell chamber (8.0 µm, 24-well, Corning, USA). A 200 µl cell suspension containing 1 × 10^4^ MΦs was inoculated into the upper chamber, and 600 µl of NPC supernatant was added to the lower chamber. This migration system was cultured at 37 °C in 5% CO_2_ for 24 h. Then, the cells that remained on the upper side of the membrane were removed by a cotton swab, and crystal violet staining solution (Beyotime, China) was used to stain the cells according to the protocol provided by the manufacturer. Finally, the stained cells were observed and imaged using an Olympus BX51 microscope (Olympus, Japan). ImageJ 1.53 (https://imagej.net/Fiji) was used to quantitatively analyze the migrated cells.

### ChIP‒qPCR

An enzymatic chromatin IP kit (9003, CST, USA) was used to extract chromatin and DNA according to the manufacturer’s protocol. ChIP grade anti-ATF3 antibody (1 µg/6 µg chromatin, ab254268, Abcam, USA) was used to precipitate chromatin bound to the ATF3 protein. Subsequent qPCR was performed as described previously, and the results are expressed as a percentage of the input. The primers were designed with the assistance of ATF3-related ChIP-seq datasets from the Cistrome Data Browser^27^. The primers were designed to target the promoter proximal region with the highest ChIP-seq peaks (Supplementary Table 2).

### siRNA-mediated gene knockdown

Transient transfection of NPCs was performed using Lipo3000 (Invitrogen, USA) and siRNA (GenePharma, China). All processes followed the manufacturer’s standard operating procedures (siRNA: lipo3000 = 20:1 (pmol:µl)). The effect of gene knockdown was confirmed by WB analysis. The siRNA sequences are shown in the supplementary materials (Supplementary Table 3).

### Data processing of public datasets

Bulk RNA-seq datasets (GSE167199, GSE176205) were obtained from the GEO database (https://www.ncbi.nlm.nih.gov/geo/). Data preprocessing was based on pipelines provided by Galaxy (http://galaxyproject.org). Fastp (v0.23.2, http://opengene.org/fastp/) and Salmon (v1.9.0, https://github.com/COMBINE-lab/Salmon) were used for pruning and to quantify the FASTA files using default parameters (Supplementary Table 4). The R packages Tximport^28^ and DESeq2^29^ were used to generate a gene expression matrix and calculate differentially expressed genes between degenerative and nondegenerative NP samples. The immune cell score was generated from the TPM matrix based on the default 64 immune cell set by using the xCell algorithm^30^. Statistical analysis of MΦ proportions was performed by an unpaired two-tailed *t*-test.

ScRNA-seq datasets (GSE165722, GSE199866) were also derived from the GEO database. The R package Seurat v4.2.0^31^ was used to analyze the scRNA-seq data. After creating Seurat objects from GSE165722 and GSE199866, we filtered out cells with fewer than 600 genes and more than 10% mitochondrial genes and then used the LogNormalize and CCA methods to integrate the standardized samples based on 2000 hypervariable genes. The integrated Seurat object was reduced and visualized by t-distributed stochastic neighbor embedding (t-SNE). Based on the Human Primary Cell Atlas (HPCA), the R package SingleR^32^ was used to automatically annotate the clusters, and MΦ subgroups were further manually annotated by scType^33^ with gene markers from Cellmarker 2.0^34,35^ (Supplementary Table 5). *T*-tests were used to compare the proportions of MΦs in different samples.

### Transcriptome sequencing and analysis

Total RNA was extracted with TRIzol reagent (Invitrogen, USA). The RNA samples were processed with the Illumina TruSeqTM RNA Sample Prep Kit to construct the library, which was then sequenced on an Illumina NovaSeq 6000 sequencer. Next, FASTP software was used for quality control and to prune the raw data. Samples were aligned to the hg19 human reference genome using HISAT2 (http://ccb.jhu.edu/software/hisat2/index.shtml) and StringTie (https://ccb.jhu.edu/software/stringtie/). RNA expression levels were quantified using RSEM (http://deweylab.github.io/RSEM/). Subsequent differential expression analysis was performed with DESeq2. The prediction of transcription factors (TFs) of differentially expressed genes was completed by the ChEA3^36^ database, and the binding motifs of TFs were determined by JASPAR^37^.

### Construction of *CCR2*^*fl/fl*^*Lyz2-cre* mice

*CCR2*^*fl/+*^ mice (No. T005892) were purchased from GemPharmatech (Nanjing, China), and the *Lyz2-cre* mouse (JAX:004781) strain was purchased from JAX®MICE (USA). All mice had a C57BL/6 genetic background. *CCR2*^*fl/+*^ mice were first crossed with *Lyz2-Cre* mice to generate F1 *CCR2*^*fl/+*^*Lyz2-Cre* mice. *CCR2*^*fl/+*^ mice were inbred to obtain *CCR2*^*fl/fl*^ mice that were then crossed with *CCR2*^*fl/+*^*Lyz2-cre* mice to generate *CCR2*^*fl/fl*^*Lyz2-cre* mice, which were macrophage-specific *CCR2*-knockout mice. *CCR2*^*fl/fl*^ mice were used as wild-type controls. Six- to eight-week-old male mice were used for further experiments. The mice were bred and maintained in specific pathogen-free cages. The sample size was based on empirical data from pilot experiments. All experimental protocols were performed according to the instructional guidelines of the China Council on Animal Care with ethics approval provided by the Institutional Animal Care and Use Committee at HUST.

### Animal experiments

Ethics approval was provided by the Institutional Animal Care and Use Committee at HUST ([2020] IACUC Number 3191), and the rat IDD model was established by caudal puncture as previously described^38^. Thirty-six 8-week-old male SD rats were purchased from the Experimental Animal Center of Tongji Medical College at HUST and were fed in the SPF Experimental Animal Center of Tongji Medical College. The rats were anesthetized with 0.3% pentobarbital sodium (40 mg/kg). After the corneal reflex disappeared completely, the caudal IVD tissue was palpated to locate the Co5/6 IVD, and then the tail diameter of the IVD was measured with a ruler. The IVD was semi-transversely punctured with a 22-gauge needle, rotated 720° and maintained for 30 s before being pulled out. One week after needle puncture, 2 µl of drug was injected into the IVD with a 29-gauge Hamilton syringe (Hamilton, USA) in the first and third weeks. In the first/second/fourth week, the rats were sacrificed and radiological and histological examinations were performed.

The mice were anesthetized with 0.3% pentobarbital sodium (40 mg/kg) by intraperitoneal administration. Then, the skin was cleaned with betadine. Tail Co 4/5 IVDs were semi-transversely punctured by a 26-gauge needle that was controlled by locking forceps clamped 3 mm from the needle tip. The needle was rotated 720° and maintained for 30 s before being pulled out. In the fourth week, the mice were sacrificed and histological examinations were performed.

### Magnetic resonance imaging (MRI)

The rats were placed on the examination bed in the prone position, and their tails were fixed on the vertical axis and kept horizontal. The sagittal section of T2-weighted imaging (T2WI) was obtained by a 3.0 T MRI scanner (Skyra 3.0 T, Siemens, Germany). The parameters were set as follows: spin echo time 1500 ms; field of view 14 cm × 14 cm; echo time 228 ms; and fault thickness 0.8 mm. Human MRI data were collected during diagnostic procedures, and all participants provided written informed consent and anonymized personal information. Syngo fastView (Siemens, Germany) was used to analyze and export the MRI images. Three spine experts who were blinded to this study graded the discs independently according to the Pfirrmann MRI-grade system. T2WI signaling intensity was measured by ImageJ 1.53 (https://imagej.net/Fiji). The hydration of disc tissues was evaluated as follows: relative water Content=$\frac{{D}_{s}}{{D}_{0}}$ (D_s_, T2-weighted intensity of discs after surgery; D_o_, T2-weighted intensity of intact discs).

### Statistical analysis and graphing software

Statistical analyses and plotting were performed using ImageJ 1.53 (https://imagej.net/Fiji), Prism v 9.0.2 software (https://www.graphpad.com/scientific-software/prism/), R (4.2.1, https://www.R-project.org) and RStudio (1.4.1717, http://www.rstudio.com/). The statistical methods are detailed in each figure. Experiments were performed at least 3 times independently, and the data are presented as the means ± SDs (*p < 0.05; **p < 0.01; ***p < 0.001; ****p < 0.0001; ns: not significant).

Other methods are described in the supplementary materials.

## Results

### Increased M1-type MΦ infiltration in degenerated NP tissue

**Fig. 1:**
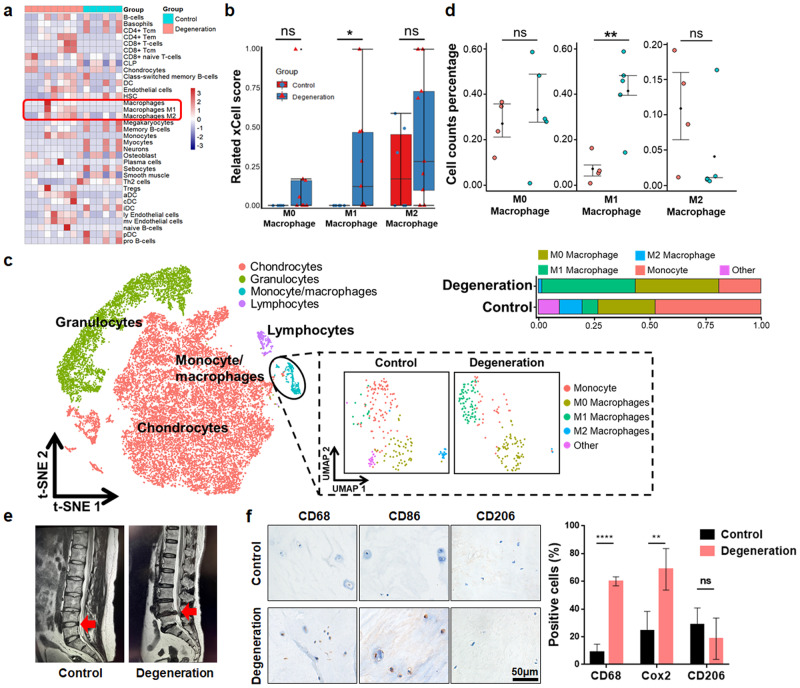
The proportion of M1 MΦs is increased in degenerated NP tissue. **a** Heatmap showing immune cell abundance in bulk RNA-seq datasets (GSE167199; GSE176205). **b** Quantitative analysis of related MΦs using the xCell score. **c** T-SNE analysis plot of single-cell transcriptome datasets (GSE165722; GSE199866) identified by SingleR. MΦ subpopulations are annotated by Sctype based on annotated marker genes. **d** Quantitative analysis of MΦ percentages in scRNA datasets. **e** MRI images of IDD patient samples. The red arrow indicates the surgical site. **f** Immunohistochemical staining of MΦ markers (CD68, CD86, CD206) in control and degenerated human NP tissue. Experiments were performed at least 3 times, and the data are presented as the means ± SDs. *p < 0.05; **p < 0.01; ***p < 0.001; ns, not significant, with Student’s *t* test.

To validate the correlation between IDD and the infiltration/polarization of MΦs, we analyzed GEO datasets (GSE167199; GSE176205) containing the RNA expression profiles of human degenerative and nondegenerative NP tissues. We found that compared with that in normal tissues, the degree of MΦ infiltration in degenerated NP tissues was significantly increased, especially that of M1-type MΦs (Fig. 1a, b). Then, SingleR was used to annotate the integrated NP single-cell RNA sequencing (scRNA-seq) data^32^. We found that cells in the NP region exhibited a monocyte-MΦ phenotype (Fig. 1c). Furthermore, we annotated and quantified the monocyte-MΦ subpopulation by the ScType method^33^, and the proportion of M1 MΦs was significantly higher in degenerative samples than in nondegenerative samples (Fig. 1c, d). Finally, the expression of MΦ-related markers was detected in degenerated (Pfirrmann grade>II) and control (Pfirrmann grade I-II) human NP tissues (Fig. 1e, Supplementary Table 6). The results showed that the expression of the MΦ marker (CD68) and M1 marker (CD86) in degenerated NP tissues was significantly increased (Fig. 1f). Therefore, MΦ infiltration in degenerative NP tissues is increased, and M1-type MΦs are dominant.

### Degenerated NPCs induce infiltration and promote the polarization of MΦs through the CCL2/7-CCR2 axis

**Fig. 2:**
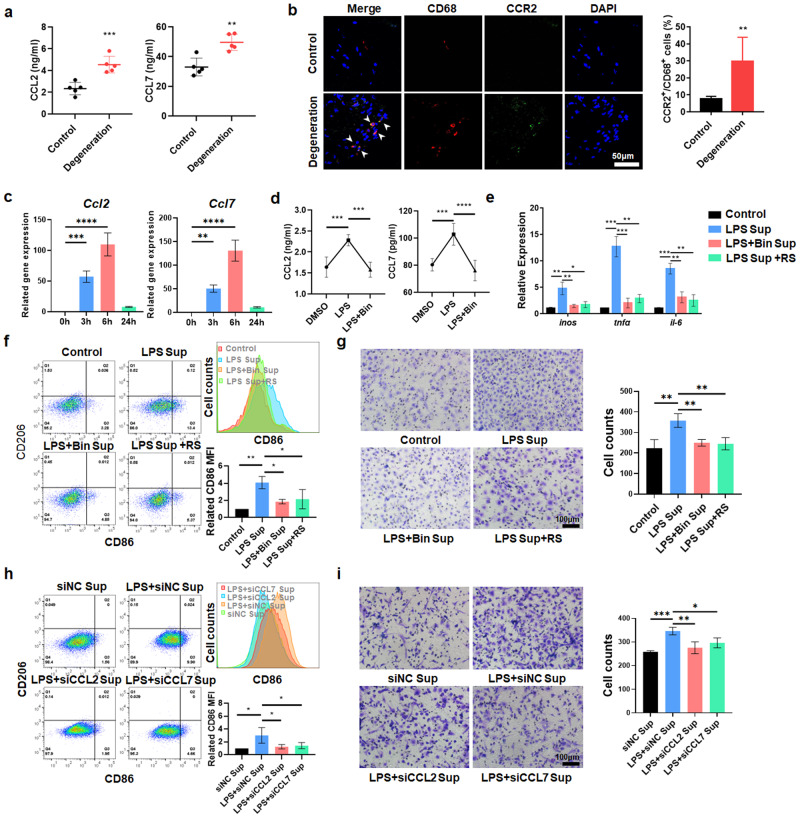
Degenerative NPCs induce the recruitment and proinflammatory polarization of MΦs through the CCL2/7-CCR2 axis. **a** ELISA analysis of the levels of CCL2 and CCL7 in human NP tissue. **b** Immunofluorescence results showing the distribution of CCR2^+^ and CD68^+^ cells in human NP tissues. **c** qPCR results showing *Ccl2* and *Ccl7* gene expression in rNPCs treated with LPS (1 µg/ml) for 0/3/6/24 h. **d** ELISA results showing the levels of CCL2 and CCL7 in the supernatant of rNPCs treated with bindarit for 1 h and LPS for 6 h. **e** qPCR results showing inflammation-related genes in rMΦs induced by supernatant from rNPCs treated with bindarit for 1 h and LPS for 6 h. **f** FCM analysis and quantification of CD68^+^CD11b^+^CD86^+^ and CD68^+^CD11b^+^CD206^+^ rMΦs as determined by geomean fluorescence intensity (GFI). The supernatant was extracted from rNPCs that were pretreated with bindarit for 1 h and LPS for 6 h with or without RS102895 for 1 h. **g** Transwell assays were used to detect the migration of rMΦs in the different groups. **h** rMΦs were treated with the supernatant extracted from LPS-treated rNPCs with CCL2 and CCL7 knockdown. The quantification of CD68^+^CD11b^+^CD86^+^ and CD68^+^CD11b^+^CD206^+^ rMΦs was detected by FCM. **i** Transwell assays were used to detect the cell migration of rMΦs in the different groups. Experiments were performed at least 3 times, and the data are presented as the means ± SDs. *p < 0.05; **p < 0.01; ***p < 0.001; ns, not significant, ANOVA. Con, control; Sup, supernatant; L, LPS; Bin, bindarit; RS, RS102895.

To determine the mechanism of MΦ recruitment and polarization, we detected the levels of the chemokines CCL2 and CCL7 in NP tissue and found that CCL2 and CCL7 were highly expressed in degenerated NP tissue (Fig. 2a). Previous studies have shown that CCL2 and CCL7 share the common receptor CCR2, which plays an important role in monocyte chemotaxis. However, there is no unified conclusion about the impact of these chemokines on MΦ polarization^39^. The immunofluorescence (IF) results showed that the percentage of CCR2^+^CD68^+^ cells was significantly increased in degenerative human NP tissues (Fig. 2b). Then, LPS was used to establish an in vitro inflammatory IDD model^24,25,40^. After 6 h of LPS exposure, the expression of CCL2 and CCL7 in rat NPCs (rNPCs) peaked. Thus, 6 h was chosen as the application time for further experiments (Fig. 2c). ELISA experiments confirmed that in response to LPS stimulation, the levels of CCL2 and CCL7 in rNPC supernatant were significantly increased, and the CCL2/7-specific antagonist bindarit decreased the levels of CCL2 and CCL7 in the supernatant (Fig. 2d). The supernatant of rNPCs was collected and cultured with rat bone marrow MΦs (rMΦs). The M1 markers *inos, tnf-α, il-6*, and CD86 were upregulated by supernatant derived from rNPCs that were pretreated with LPS, and bindarit reversed this effect (Fig. 2e, f). Moreover, RS102895, a CCR2-specific antagonist, was administered to rMΦs exposed to LPS-pretreated rNPC supernatant. We found that inhibiting CCR2 in rMΦs reversed the proinflammatory effects of degenerated rNPC supernatant (Fig. 2e, f). Transwell assays also showed that degenerated rNPC supernatant effectively promoted rMΦ migration, which was significantly inhibited by bindarit and RS102895 (Fig. 2g). We knocked down CCL2 and CCL7 in rNPCs to decrease the levels of CCL2 and CCL7 in the supernatant (Supplementary Fig. 1). Flow cytometry (FCM) and Transwell assays showed that knockdown of CCL2 and CCL7 effectively reduced the expression of the M1 marker (CD86) and the migration of MΦs (Fig. 2h, i). However, the change in the M2 marker (CD206) was not significant (Supplementary Fig. 2).

**Fig. 3:**
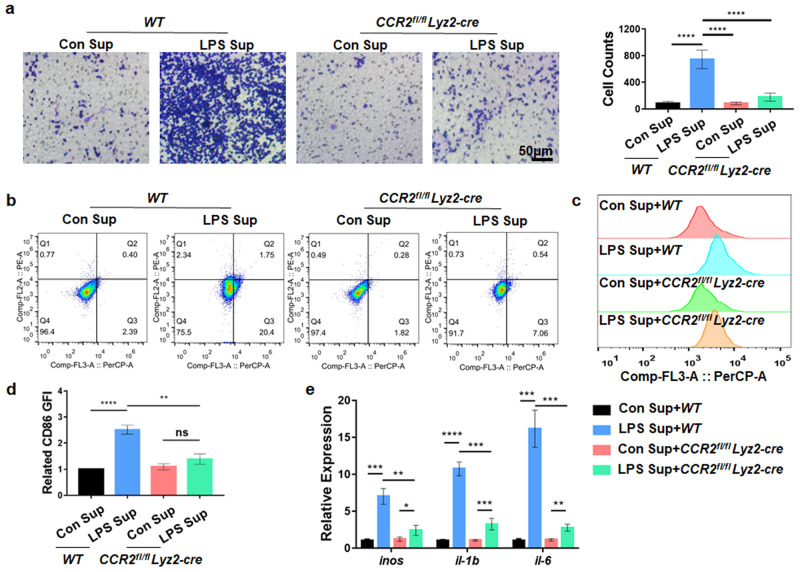
Deletion of CCR2 inhibits MΦ infiltration and proinflammatory polarization. **a** After mMΦs from *WT* or *CCR2*^*fl/fl*^*Lyz2-cre* mice were treated with LPS-induced mNPC supernatant for 24 h, the migration of mMΦs was determined by Transwell assays. **b** CD68^+^CD86^+^ and CD68^+^CD206^+^ mMΦs were detected by FCM. **c**, **d** Quantification of CD68^+^CD86^+^ mMΦs by GFI. **e** qPCR results showing inflammation-related genes in *CCR2*^*fl/fl*^ *Lyz2-cre* mMΦs treated with supernatant extracted from mNPCs treated with LPS for 6 h. Experiments were performed at least 3 times, and the data are presented as the means ± SDs. *p < 0.05; **p < 0.01; ***p < 0.001; ****p < 0.0001; ns, not significant, with ANOVA. Con, control; Sup, supernatant.

To further explore the specific role of the CCL2/7-CCR2 axis, we generated *CCR2*^*fl/fl*^*Lyz2-cre* mice by crossing *Lyz2-cre* mice with *CCR2* ^*fl/fl*^ mice and treated MΦs from *CCR2*^*fl/fl*^*Lyz2-cre* and *WT* mice (mMΦs) with supernatant derived from LPS-induced degenerative mouse NPCs (mNPCs). We found that the number of migrated *CCR2*^*fl/fl*^*Lyz2-cre* mMΦs was significantly decreased compared with that of *WT* mMΦs treated with the supernatant of LPS-pretreated mNPCs (Fig. 3a). FCM showed that knockout of CCR2 reduced the proportion of CD86^+^ MΦs in the *CCR2*^*fl/fl*^ *Lyz2-cre* group (Fig. 3b–d). In addition, *CCR2*^*fl/fl*^*Lyz2-cre* mMΦs exhibited downregulated expression of *inos*, *il-1b*, and *il-6* compared with *WT* mMΦs when treated with the supernatant from LPS-pretreated mNPCs (Fig. 3e). These results suggest that degenerated NPCs recruit and promote the proinflammatory MΦ phenotype through the CCL2/7-CCR2 axis.

### The ISR participates in CCL2/7 expression in degenerated NPCs

**Fig. 4:**
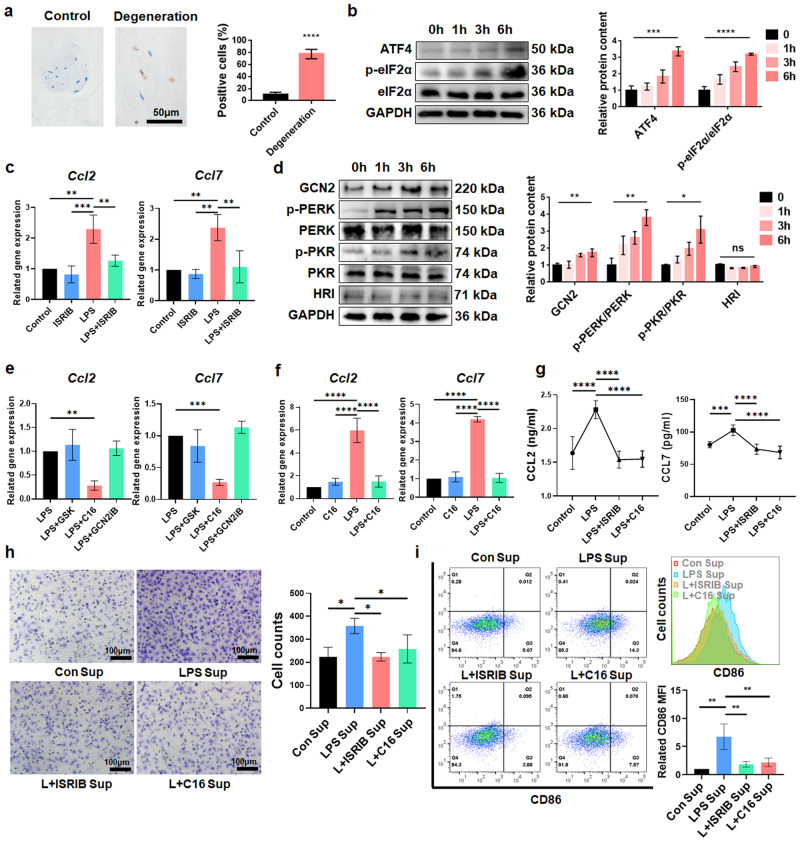
PKR-mediated ISR is involved in the recruitment and polarization of MΦs during IDD. **a** Representative IHC results showing p-eIF2α in degenerated and nondegenerated human NP tissues. **b** The protein expression of ATF4, p-eIF2α and eIF2α in rNPCs treated with LPS for 6 h was detected by WB analysis. **c** qPCR results showing *CCL2* and *CCL7* gene expression in rNPCs after ISRIB pretreatment for 1 h and LPS treatment for 6 h. **d** The protein expression of ISR-related kinases in rNPCs treated with LPS for 0/1/3/6 h. **e** qPCR results showing the expression of *CCL2* and *CCL7* in rNPCs after GSK2606414, PKR-IN-C16 or GCN2iB pretreatment for 1 h and LPS treatment for 6 h. **f** qPCR results showing *CCL2* and *CCL7* expression in rNPCs after PKR-IN-C16 pretreatment for 1 h and LPS treatment for 6 h. **g** ELISA results showing the levels of CCL2 and CCL7 in the supernatant from rNPCs after ISRIB or C16 pretreatment for 1 h and LPS treatment for 6 h. **h** Transwell assays were used to detect the migration of rMΦs. rMΦs were treated with supernatant extracted from rNPCs that were pretreated with C16 or ISRIB for 1 h and LPS for 6 h. **i** FCM analysis and quantification of CD68^+^CD11b^+^CD86^+^ or CD68^+^CD11b^+^CD206^+^ rMΦs. Experiments were performed at least 3 times, and the data are presented as the means ± SDs. *p < 0.05; **p < 0.01; ***p < 0.001; ****p < 0.0001; ns, not significant, with ANOVA. Con, control; Sup, supernatant; L, LPS; Bin, bindarit; C16, PKR-IN-C16; GSK, GSK2606414.

The hallmark of ISR activation is eIF2α phosphorylation at Ser-51^14^. Thus, we evaluated the expression of p-eIF2α in non-degenerated and degenerated NP tissues and found that the percentage of p-eIF2α-positive cells was significantly increased in degenerated human NP tissues (Fig. 4a), indicating that the ISR may be activated in IDD. The levels of ATF4 and p-eIF2α/eIF2α in rNPCs were increased after LPS treatment, and this change was most significant at 6 h, indicating that the ISR was activated in an inflammatory environment (Fig. 4b). We used ISRIB as a specific inhibitor of ISR and found that ISRIB reduced the LPS-induced expression of *Ccl2* and *Ccl7* (Fig. 4c), indicating that the ISR mediated the expression of CCL2 and CCL7 in NPCs. GCN2, PKR, PERK, and HRI^14^ are upstream kinases that mediate ISR activation. We found that the levels of GCN2, p-PKR/PKR, and p-PERK/PERK increased with the time of LPS treatment, but there was no obvious dimerization or expression changes in HRI (Fig. 4d). Therefore, GCN2, PKR, and PERK may mediate ISR activation in degenerated NPCs. Then, we used GCN2iB, PKR-IN-C16, and GSK2606414 to specifically inhibit GCN2, PKR, and PERK, respectively, and inhibiting PKR phosphorylation with C16 reversed the effects of the ISR on inducing the expression of *Ccl2* and *Ccl7*; however, the effects of the other inhibitors were not significant (Fig. 4e, f). These results were confirmed by ELISA (Fig. 4g). Then, the effects of ISR activation in NPCs on MΦ recruitment and polarization were evaluated. Transwell assays and FCM showed that after rNPCs were treated with C16 and ISRIB, MΦ migration was significantly reduced, and the expression of the M1 marker CD86 (but not CD206) was significantly downregulated (Fig. 4h, i, Supplementary Fig. 3). These results indicate that PKR-mediated ISR activation regulates the production of CCL2/7 in NPCs, which ultimately induces MΦ recruitment and proinflammatory polarization.

### ATF3 regulates CCL2/7 transcription under ISR activation

**Fig. 5:**
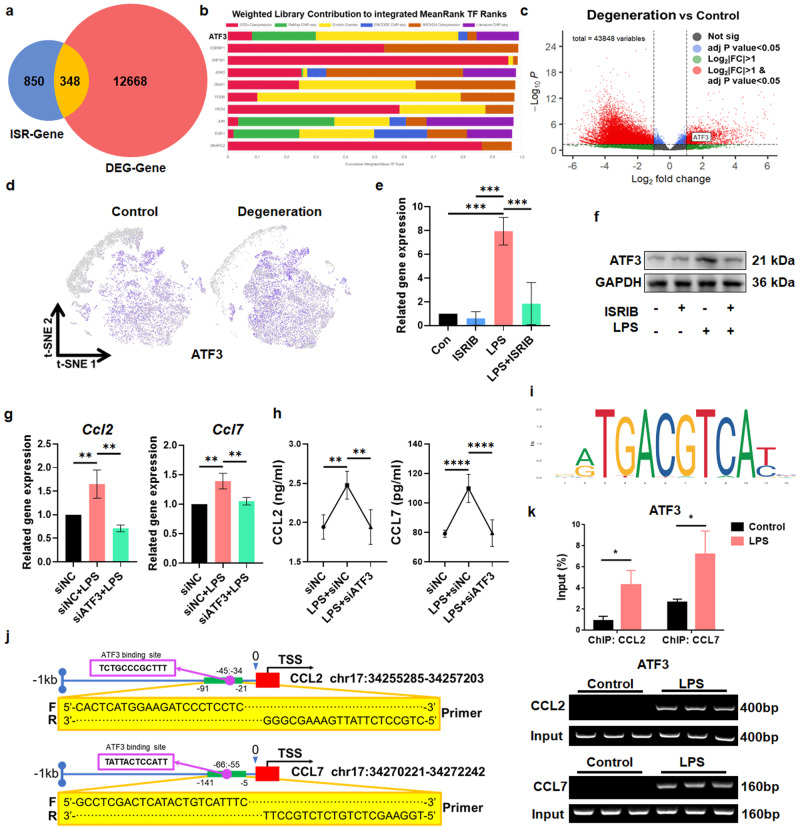
ATF3 is the key regulator of CCL2/7 in IDD. **a** Venn diagram showing overlapping genes based on ISR-genes and DEG-genes. **b** The CheA3 database was used to predict the transcription factors of the overlapping genes obtained in (A). **c** The volcano plot of DEG genes showed that the ATF3 gene was significantly upregulated in degenerative human NP tissue. **d** Relative expression of ATF3 in all cells projected onto a t-SNE plot based on the scRNA-seq data. **e** qPCR analysis of the expression of *Atf3* in rNPCs treated with ISRIB for 1 h and LPS for 6 h. **f** Protein expression of ATF3 in rNPCs in the different groups. **g** qPCR analysis of *Ccl2* and *Ccl7* gene expression in LPS-treated rNPCs with ATF3 knockdown. **h** ELISA analysis of CCL2 and CCL7 levels in the supernatant of rNPCs. **i** The ATF3 binding motif was obtained from JASPAR. **j** Schematic illustration of the location of primers used for ChIP-qPCR. **k** ChIP analysis of control and LPS-treated rNPCs using ATF3 antibodies at the *Ccl2* and *Ccl7* promoters. Fold changes are expressed as the percentage of input. Experiments were performed at least 3 times, and the data are presented as the means ± SDs. *p < 0.05; **p < 0.01; ***p < 0.001; ****p < 0.0001; ns, not significant, with ANOVA.

To explore the mechanism by which the ISR induces CCL2/7 expression, we exposed rNPCs to LPS for 6 h after pretreatment with or without ISRIB and then performed bulk RNA-seq and differential expression analysis. Using *p* < 0.05 and |Log2FC| > 1 as screening criteria, we identified differentially expressed ISR-related genes (ISR-genes) (Supplementary Fig. 4). Then, differential expression in the integrated GEO transcriptome datasets, including GSE167199 and GSE176205, were analyzed, and the differentially expressed genes of degenerated and non-degenerated human NP tissues (DEG-genes) were obtained. The intersection of ISR-genes and DEG-genes revealed 348 overlapping differentially expressed genes (Fig. 5a, Supplementary Table 7). Then, the ChEA3 database was used for transcription factor prediction. We found that ATF3 played a leading role among the predicted TFs (Fig. 5b) and was closely related to other TFs (Supplementary Fig. 5). We also found that ATF3 was highly expressed in degenerated NP tissues (Fig. 5c). Analysis of the scRNA-seq datasets (GSE165722, GSE199866) showed that ATF3 was mainly expressed in chondrocyte-like cells, and the expression of ATF3 in degenerative tissues was increased (Fig. 5d). Consistently, LPS induced the expression of ATF3 in NPCs, and this effect was inhibited by ISRIB (Fig. 5e, f), indicating that ATF3 was regulated by the ISR in degenerated NPCs. Subsequently, we knocked down the expression of ATF3 (Supplementary Fig. 6) to verify its role in regulating CCL2 and CCL7 and found that the levels of CCL2 and CCL7 in rNPCs and their supernatant decreased significantly (Fig. 5g, h). ChIP‒qPCR analysis showed that after LPS treatment, the occupancy of ATF3 near the *Ccl2/7* promoters was significantly increased, indicating that ATF3 directly bound to the *Ccl2*/*7* promoters to regulate their expression (Fig. 5i–k). Therefore, the ISR promotes the expression of ATF3 to increase the transcription of CCL2/7.

### The TLR4-mtROS-dsRNA pathway mediates ISR activation

**Fig. 6:**
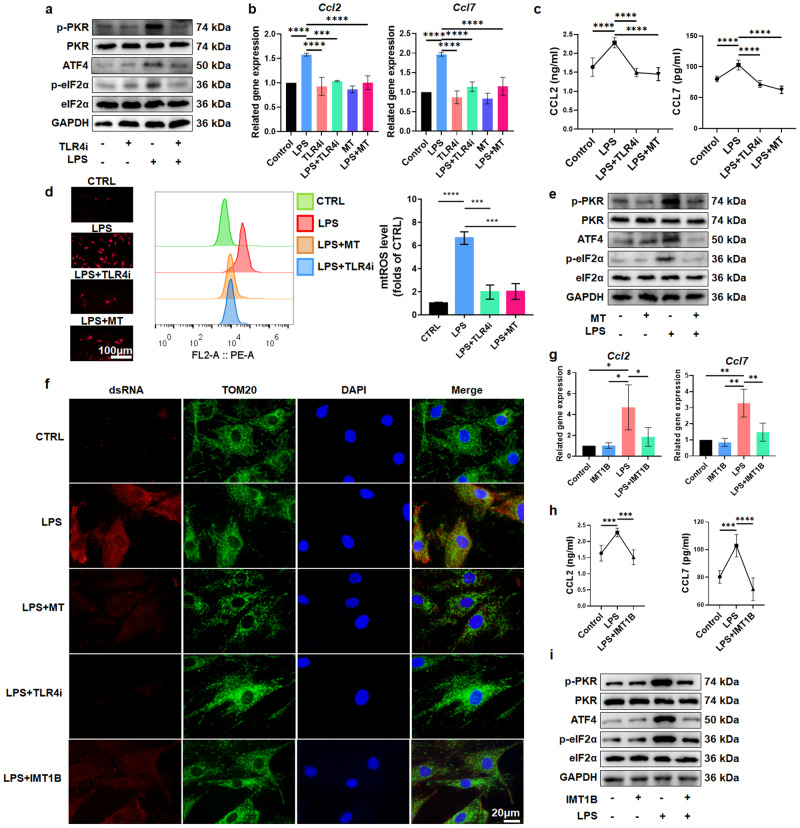
LPS induces PKR-mediated ISR activation and CCL2/7 release through the TLR4-mtROS-dsRNA pathway. **a** The expression of PKR-ISR pathway-related proteins in rNPCs with or without TLR4-IN-C34 pretreatment for 1 h and LPS treatment for 6 h. **b** *Ccl2* and *Ccl7* gene expression in rNPCs with or without TLR4-IN-C34 or MitoTempo pretreatment for 1 h and LPS treatment for 6 h. **c** ELISA results showing CCL2 and CCL7 levels in supernatants derived from rNPCs with or without TLR4-IN-C34 or MitoTempo pretreatment for 1 h and LPS treatment for 6 h. **d** Fluorescence staining and FCM analysis of mtROS by MitoSOX in rNPCs pretreated with TLR4-IN-C34 or MitoTempo for 1 h and then treated with LPS for 6 h. **e** PKR-ISR pathway-related protein expression in rNPCs with or without MitoTempo pretreatment for 1 h and LPS treatment for 6 h. **f** Immunofluorescence staining of dsRNA and mitochondria in rNPCs pretreated with TLR4-IN-C34, MitoTempo or IMT1B for 1 h and then treated with LPS for 6 h. **g** *Ccl2* and *Ccl7* gene expression in rNPCs with or without IMT1B pretreatment for 1 h and LPS treatment for 6 h. **h** ELISA results showing CCL2 and CCL7 levels in rNPC supernatant. **i** The expression of PKR-ISR pathway-related proteins in rNPCs. Experiments were performed 3 times, and the data are presented as the means ± SDs. *p < 0.05; **p < 0.01; ***p < 0.001; ****p < 0.0001; ns, not significant, with ANOVA. Con, control; MT, MitoTempo; TLR4i, TLR4-IN-C34.

Toll-like receptor 4 (TLR4), which is a cell surface receptor, senses proinflammatory substances in the external environment and can regulate the expression of cytokines in IDD^41^. Previous studies have reported that TLR4 mediates sepsis-induced liver damage through the ISR^42^. Therefore, we hypothesized that ISR activation in degenerated NPCs may be related to TLR4. The application of TLR4-IN-C34 (a specific inhibitor of TLR4) significantly reduced the levels of p-PKR/PKR, p-eIF2α/eIF2α, and ATF4 (Fig. 6a, Supplementary Fig. 7a). PCR and ELISA showed that blocking TLR4 effectively inhibited the expression of CCL2 and CCL7 and their accumulation in the supernatant (Fig. 6b, c), indicating that TLR4 mediates LPS-induced activation of the ISR and promotes the production of CCL2 and CCL7.

Mitochondrial ROS are important signaling molecules in the cellular stress network^43^. TLR4 activation can cause the accumulation of mtROS in cells during innate immune responses^44^. We found that the TLR4 inhibitor decreased the cellular levels of mtROS (Fig. 6d), indicating that the production of mtROS in NPCs was regulated by TLR4. MitoTempo was used to consume mtROS in NPCs (Fig. 6d), and we found that MitoTempo effectively reduced the levels of p-PKR/PKR, p-eIF2α/eIF2α, and ATF4 in rNPCs (Fig. 6e and Supplementary Fig. 7b). Moreover, MitoTempo downregulated the expression of CCL2 and CCL7 and reduced their levels in the supernatant (Fig. 6b, c). These results suggest that in response to LPS stimulation, TLR4 promotes activation of the ISR by inducing mtROS production, thus mediating the production of CCL2/7.

We previously identified that PKR was responsible for ISR activation in degenerated NPCs. In response to cell stress induced by astrosporin or okadaic acid, dsRNA accumulates in mitochondria and causes PKR activation^45^. In chondrocytes, mitochondrial oxidative stress leads to a cell stress response mediated by dsRNA^46^. Therefore, dsRNA may be the intermediate stimulus of mtROS that regulates the ISR in an inflammatory environment. We used J2 antibody to detect endogenous dsRNA and found that dsRNA significantly accumulated after LPS treatment, and inhibiting TLR4 and mtROS reduced dsRNA levels, indicating that TLR4/mtROS mediated LPS-induced dsRNA accumulation (Fig. 6f). IMT1B is a specific inhibitor of POLRMT that prevents the generation of mitochondrial dsRNA by inhibiting mitochondrial mRNA transcription^45,47^. We found that IMT1B effectively reduced dsRNA levels (Fig. 6f). In addition, IMT1B reduced the levels of CCL2/7 in cells and supernatants and downregulated the expression of p-PKR/PKR, p-eIF2α/eIF2α and ATF4 (Fig. 6g–i, and Supplementary Fig. 7c), suggesting that dsRNA, which is a downstream signaling molecule of TLR4 and mtROS, mediates the activation of PKR and the ISR, thus leading to the synthesis and release of CCL2/7.

### Inhibiting the CCL2/7-CCR2 axis and ISR alleviates the proinflammatory microenvironment in IDD

**Fig. 7:**
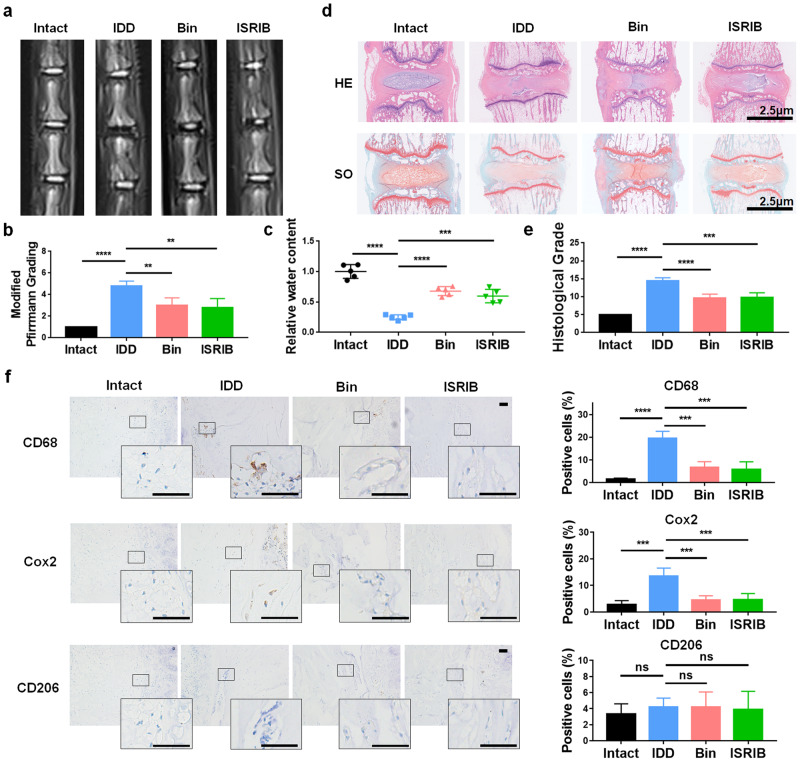
Inhibiting the ISR and blocking the CCL2/7-CCR2 axis ameliorates IDD in vivo. **a** MRI images (T2WI) of rat caudal vertebrae after 4 weeks of modeling. **b** The degenerative grades of IVDs were evaluated according to the Pfirrmann grade based on T2WI images. **c** Quantitative comparison of the water content of IVD tissues in the different groups. **d** HE and SO staining of the operated segments. **e** The degenerative grades based on the histological images. **f** Representative immunohistochemical images and quantitative analysis of CD68, COX2 and CD206 in each group of rat IVD tissues. Experiments were performed 5 times independently, and the data are presented as the means ± SDs. *p < 0.05; **p < 0.01; ***p < 0.001; ****p < 0.0001; ns, not significant, with ANOVA. Bin, bindarit.

A rat IDD model was established by 22-gauge needle puncture of Co5/6. One week after needle puncture, normal saline (0.9%, 2 μL), bindarit (300 µM, 2 μL) or ISRIB (2 µM, 2 μL) was injected into the injured discs using 29-gauge needles and microsyringes. Four weeks later, rat tails were collected for radiological and histological analysis. MRI images were obtained and modified Pfrimann grading^20^ were performed to analyze the degenerative grade in each group. The results showed that the T2WI intensity in the IDD group was significantly lower than that in the intact group, and bindarit and ISRIB rescued the loss of T2WI intensity (Fig. 7a, b). Quantitative analysis of water content based on the MRI images indicated that bindarit and ISRIB preserved NP hydration (Fig. 7c).

The grading system of Han et al. was used to evaluate histological changes^48^. Based on hematoxylin-eosin staining (HE) and safranin O-fast green (SO) staining, we found that the IDD group exhibited ruptured fibers, severe cell loss, obscured borders between AF and NP tissues, irregular NP shapes, and relatively higher degenerative histological grades than the intact group, and bindarit and ISRIB improved the morphology of NP tissues and fibers to a certain extent (Fig. 7d, e). Subsequently, MΦs (CD68) and their polarization markers (M1: COX2; M2: CD206) were evaluated by immunohistochemical (IHC) staining. Quantitative analysis of the IHC data showed that more MΦs infiltrated and exhibited the M1 phenotype in rNP tissues of the IDD group, and the administration of bindarit and ISRIB significantly reversed this effect at different stages (Fig. 7f, Supplementary Fig. 8). Notably, although the M1 marker peaked on the 7th day, as time elapsed, the ratio of total MΦs to M2 MΦs increased at 7 and 14 days, and the proportion of M1 MΦs decreased. This likely reflects an incomplete healing process, which is consistent with previous studies^40,49–52^.

**Fig. 8:**
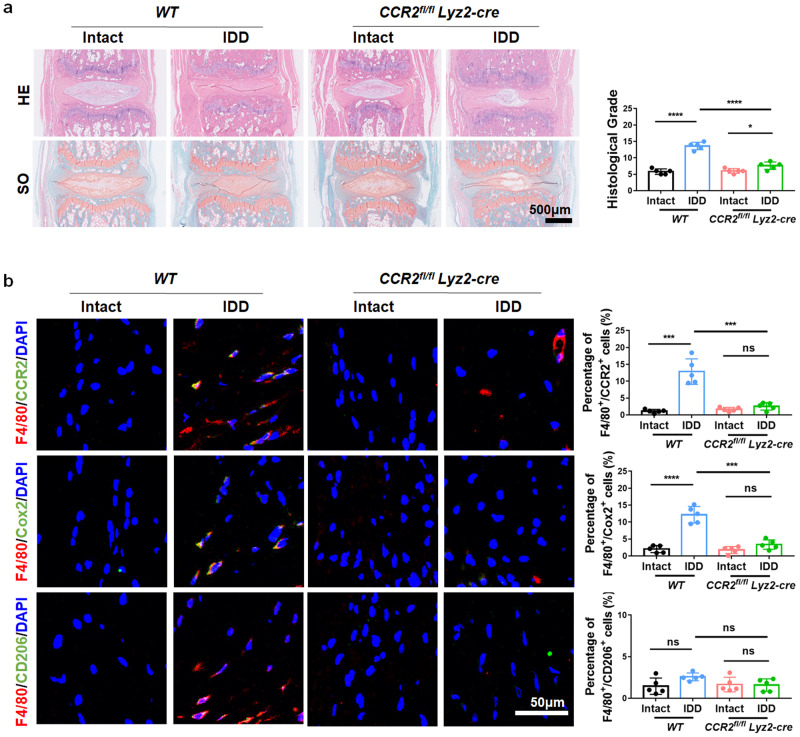
CCR2 knockout in MΦs inhibits MΦ infiltration and IDD. **a** HE and SO staining of *CCR2*^*fl/fl*^*Lyz2-cre* and *WT* mouse IVD tissue. **b** Immunofluorescence images and quantitative analysis of F4/80^+^/CCR2^+^, F4/80^+^/Cox2^+^ and F4/80^+^/CD206^+^ double-positive cells in the IVD tissue of *CCR2*^*fl/fl*^*Lyz2-cre* and *WT* mice. Experiments were performed 5 times independently, and the data are presented as the means ± SDs. *p < 0.05; **p < 0.01; ***p < 0.001; ns: not significant, ANOVA.

Furthermore, a 26-gauge needle puncture-induced mouse disc degenerative model was established in *CCR2*^*fl/fl*^*Lyz2-Cre* and *WT* mice at Co5/6. After 4 weeks, IVD tissue was collected. Histological analysis showed that the degenerative histological grade was significantly reduced in *CCR2*^*fl/fl*^*Lyz2-cre* mice (Fig. 8a), as evidenced by HE and SO staining. Immunofluorescence staining showed that F4/80^+^/Cox2^+^ double-positive cells were decreased in the discs of *CCR2*^*fl/fl*^*Lyz2-cre* mice relative to their wild-type *(WT)* littermates, indicating that knockout of *CCR2* effectively rescued disc degeneration and reduced the infiltration of MΦs in IVDs (Fig. 8b). Therefore, inhibiting ISR activation and blocking the CCL2/7-CCR2 axis is an effective strategy to alleviate inflammation and the progression of IDD in vivo.

## Discussion

The critical role of inflammation in IDD progression has recently drawn much attention^5^. Proinflammatory MΦs act primarily on the chronic inflammatory cascade of IDD^10^. Herein, we explored the mechanism of MΦ recruitment and polarization in IVD tissues. Previous studies have shown that chemokines (like CCL2) content and macrophage infiltration are increased in degenerated intervertebral discs^51,52^. In the tumor microenvironment, CCL2/7 mainly polarizes MΦs into M2-like tumor-related MΦs, and in inflammatory diseases, these factors tend to polarize MΦs toward the M1 phenotype^39,53^. However, the role of CCL2/7 in IDD has not been clearly defined. CCR2 is a typical chemokine receptor expressed by monocytes. Its binding with the ligands CCL2 and CCL7 is crucial for monocyte recruitment to inflammatory sites^54^. In IDD tissue, NPCs are the main source of the chemokines CCL2/7^6^. Therefore, we hypothesized that NPCs could induce MΦ infiltration and polarization by releasing CCL2/7. We found that the production and release of CCL2/7 was significantly increased in degenerated NPCs, and inhibiting or knocking out CCR2 or antagonizing CCL2/7 inhibited MΦ migration and proinflammatory polarization in vitro and in vivo. Therefore, the CCL2/7-CCR2 axis mediates MΦ infiltration and M1 polarization induced by degenerated NPCs, and targeting CCL2/7 is a feasible strategy to ameliorate IDD. However, the factors that trigger the production and release of CCL2/7 in degenerated NPCs remain unknown.

In the IDD microenvironment, NPCs must withstand a variety of stimuli, including inflammation, ROS, and pressure^23,55,56^. The ISR, which is an important stress response, maintains homeostasis by downregulating global protein translation and upregulating the translation of stress-related proteins such as ATF4^13^. Our study showed that the proportion of p-eIF2α-positive cells was increased in degenerated disc tissues, which was consistent with a previous study^57^, proving that the ISR was activated in IDD. Although the ISR protects against adverse stimulation, its benefits to cells and tissues vary with duration and degree^13,58^. For example, the ISR protects myocardial cells from ischemia‒reperfusion injury by reducing oxidative stress in the heart^19^, and lipid-induced ISR activation promotes atherosclerosis by promoting inflammasome activation^59^.

Previous studies have shown that the ISR can regulate inflammatory processes, such as the production of IL-8^60^ and the activation of inflammasomes^59^. We examined the regulatory effects of the ISR on CCL2/7 in NPCs in response to the inflammatory microenvironment. We found that inhibition of the ISR reduced the release of CCL2/7 from NPCs and blocked the effects of cell supernatants on promoting MΦ infiltration and inflammatory polarization. Furthermore, in vivo experiments showed that ISR deactivation reduced the infiltration of MΦs, thus alleviating the progression of IDD and suggesting the primary role of the ISR in regulating IDD inflammation.

To further explore the downstream mechanism of ISR-mediated expression of CCL2/7, we performed bioinformatics analysis and showed that ATF3 was a key transcription factor affected by the ISR. ATF3 is a member of the ATF/CREB transcription factor family, and its expression can be upregulated by hypoxia, an inflammatory environment, DNA damage and other forms of cellular stress^61^. ATF3 is mainly activated in an ISR-ATF4-dependent manner and regulates multiple genes related to cell survival and cell stress^62,63^. Our results showed that the ISR mediated the activation of ATF3 in NPCs in an inflammatory microenvironment, which further regulated CCL2/7 expression through ATF3 binding to the DNA promoter, reflecting the direct relationship between the ISR and MΦ infiltration in IDD.

ISR activation is mediated by four upstream kinases (PERK, GCN2, PKR, HRI)^18^. In this study, we found that in the inflammatory microenvironment, ISR in NPCs was mainly activated by PKR. PKR is a key sensor of human innate immunity, has two binding motifs for viral dsRNA at the N-terminus and plays an important role in the antiviral response^64^. Recently, the role of endogenous dsRNA in PKR activation has received much attention^45^. Evidence has shown that mitochondrial stress in chondrocytes leads to the accumulation of endogenous dsRNA in cells, which in turn mediates the activation of PKR and ultimately participates in the occurrence of osteoarthritis^46^. Therefore, dsRNA may mediate the regulatory effects of mtROS on ISR activation in inflammatory NPCs. Our results showed that mitochondrial dsRNA effectively promoted the activation of PKR and the ISR. Inhibiting PKR and the ISR decreased the release of CCL2/7 from NPCs and further reduced the infiltration and M1 polarization of MΦs. Thus, the ISR was activated through the PKR pathway in degenerated NPCs, and this effect was mediated by the accumulation of mitochondrial dsRNA.

We further investigated the mechanism of dsRNA accumulation in degenerated NPCs. As a cell surface receptor, TLR4 binds with a variety of pathogen-related molecular models (PAMPs) and damage-related molecular models (DAMPs) to sense different external inflammatory stimuli^65,66^. The activation of TLR4 can upregulate the level of mitochondrial ROS and further damage mitochondrial structure and function^67^. However, it is not clear whether mtROS can cause the accumulation of dsRNA. Our results showed that both TLR4 and mtROS inhibitors reduced the level of dsRNA in cells, and inhibiting TLR4 decreased the cellular accumulation of mtROS. Inhibiting TLR4, mtROS and dsRNA significantly reduced activation of the ISR and the synthesis and release of CCL2/7 in NPCs. This suggests that the TLR4-mtROS-dsRNA pathway mediates the response of NPCs to the inflammatory microenvironment by regulating ISR activity, thus affecting the synthesis and release of chemokines.

**Fig. 9:**
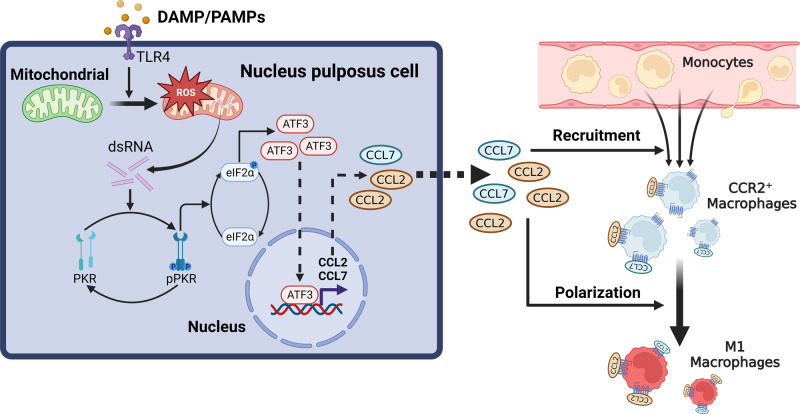
Schematic diagram of nucleus pulposus cells regulating macrophages in the intervertebral disc microenvironment. The proinflammatory environment activates the ISR in NPCs and mediates macrophage infiltration and polarization through the CCL2/7-CCR2 axis.

In conclusion, our study clarified the link between cell stress and the infiltration and polarization of MΦs in IDD. Mitochondria act as the central signal platform, inflammatory signals upregulate intracellular mtROS by activating the TLR4 receptor, and the increase in mtROS promotes the production of endogenous dsRNA, which further activates the ISR pathway through PKR and finally upregulates the expression of the chemokines CCL2/7. Then, MΦs are recruited to the degenerated IVD region and are polarized into the proinflammatory M1 phenotype through the CCL2/7-CCR2 axis, leading to the progression of IDD (Fig. 9). Blocking the ISR or CCL2/7 is a feasible approach to alleviate IDD. This study clarified the signaling pathway by which NPCs induce MΦ infiltration and proinflammatory polarization and provided potential targets for the treatment of IDD.

### Supplementary information

Supplymentary materials

## Data Availability

The data that support the findings of this study are available from the corresponding author upon reasonable request.
