# Supplementary material for: Nucleus pulposus cells regulate macrophages in degenerated intervertebral discs via the integrated stress response-mediated CCL2/7-CCR2 signaling pathway
Source: Exp Mol Med. 2024 Feb 5;56(2):408–21. doi: 10.1038/s12276-024-01168-4 (PMC10907345; doi:10.1038/s12276-024-01168-4)
Supplement: Supplementary file 1 — Supplymentary materials [file 12276_2024_1168_MOESM1_ESM.pdf]

Supplementary Table 1. qPCR primers

Gene	Primer type	Sequence
ARG1	Forward primer	AGTGGCGTTGACCTTGTCTTGT
	Reverse primer	TGGCCTGGTTCTGTTCGGTTT
ATF3	Forward primer	CGAGCGAAGACTGGAGCAAA
	Reverse primer	CCGATGGCAAAGGTGCTTGTT
CCL2	Forward primer	AGCAGCAGGTGTCCCAAAGAAG
	Reverse primer	AAGTGCTTGAGGTGGTTGTGGA
CCL7	Forward primer	GACCAATTCATCCACTTGCTGCT
	Reverse primer	AGCACAGACTTCCATGCCCTTT
CD86	Forward primer	GGTCCCAGTTCACTTCTGTGCT
	Reverse primer	AACACCACTGTCCTGCTTGGA
CD206	Forward primer	ACAAAGGGACGTTTCGGTGGA
	Reverse primer	TGGGTTCAGGAGTTGTTGTGGG
GAPDH	Forward primer	AGAGACAGCCGCATCTTCTTGT
	Reverse primer	CCAGCTTCCCATTCTCAGCCTT
IL-1β	Forward primer	CAGCTACCTATGTCTTGCCCGT
	Reverse primer	CGTCATCATCCCACGAGTCACA
IL-10	Forward primer	GGCCCAGAAATCAAGGAGCAT
	Reverse primer	TGTCACGTAGGCTTCTATGCAG
iNOS	Forward primer	AGGCTTGGGTCTTGTTAGCC
	Reverse primer	TTGTTGGGCTGGGAATAGCAC
IL-6	Forward primer	ACCACCCACAACAGACCAGTA
	Reverse primer	GGAACTCCAGAAGACCAGAGCA
TNF-α	Forward primer	ACACCATGAGCACGGAAAGCA
	Reverse primer	GCCACGAGCAGGAATGAGAAGA
Cox2	Forward primer	GCCGGGTCTGATGATGTATGCT
	Reverse primer	TGGTTTGGAACAGTCGCTCGT

Supplementary Table 2. Chip-qPCR primers

ATF3 binding site in the promoter of CCL2 gene		
**Primers**	Forward primer	CACTCATGGAAGATCCCTCCTC
	Reverse primer	CTGCCTCTTATTGAAAGCGGG
**Target DNA fragment**	>hg38_dna range=chr17:34254918-34255322 5'pad=0 3'pad=0 strand=+ repeatMasking=none<br>GCTGGCAGCGAGCCTGACATGCTTTCATCTAGTTTCCTCGCTTCCTTCCTTTTCT<br>GCAGTTTTCGCTTCAGAGAAAGCAGAATCCTTAAAAATAACCCTCTTAGTTCAC<br>ATCTGTGGTCAGTCTGGGCTTAATGGCACCCCATCCTCCCCATTTGCTCATTTGGT<br>CTCAGCAGTGAATGGAAAAAGTGTCTCGTCCTGACCCCCTGCTTCCCTTTCCTA<br>CTTCCTGGAAATCCACAGGATGCTGCATTTGCTCAGCAGATTTAACAGCCCACTT<br>ATCACTCATGGAAGATCCCTCCTCCTGCTTGACTCCGCCCTCTCTCCCTCTGCCC<br>GCTTTCAATAAGAGGCAGAGACAGCAGCCAGAGGACCGAGAGGCTGAGACTAA<br>CCCAGAAACATCCAATTCTCAA	

ATF3 binding site in the promoter of CCL7 gene		
**Primers**	Forward primer	GCCTCGACTCATACTGTCATTTC
	Reverse primer	TGGAAGCTCTGTCTCTGCCTT
**Target DNA fragment**	>hg38_dna range=chr17:34270073-34270232 5'pad=0 3'pad=0 strand=+ repeatMasking=none<br>CAGGCTAGCCTCGACTCATACTGTCATTTCCTATCCTCCCACTGAAGTGCACTGG<br>CTCAGCAGATTTATTACTCCATAGATTTATTACTCCATTCTATGATTCATCCTCTCT<br>GCTTCCTATAAAAGGCAGAGACAGAGCTTCCAGAGGAGCAGAGGGGCT	

Supplementary Table 3. siRNA sequences

siRNA	Type	Sequence
si-CCL2	Sense	GGACUUCAGCACCUUUGAATT
	Antisense	UUCAAAGGUGCUGAAGUCCTT
si-CCL7	Sense	GUCCCUGGGAAGCUGUUAUTT
	Antisense	AUAACAGCUUCCCAGGGACTT
si-ATF3	Sense	GAGUCAGUCACCAUCAACATT
	Antisense	UGUUGAUGGUGACUGACUCTT

Supplementary Table 4. Fastp and Salmon parameters on Galaxy platform

1. Fastp Tool Parameters

Input Parameter	Value
Single-end or paired reads	paired_collection
Select paired collection(s)	No dataset collection.
adapter_trimming_options	
Disable adapter trimming	False
Adapter sequence for input 1	Empty.
Adapter sequence for input 2	Empty.
global_trimming_options	
Trim front for input 1	Not available.
Trim tail for input 1	Not available.
Trim front for input 2	Not available.
Trim tail for input 2	Not available.
overrepresented_sequence_analysis	
Enable overrepresented analysis	False
Overrepresentation sampling	Not available.
filter_options	
quality_filtering_options	
Disable quality filtering	False
Qualified quality phred	Not available.
Unqualified percent limit	Not available.
N base limit	Not available.
length_filtering_options	
Disable length filtering	False
Length required	Not available.
Maximum length	Not available.
low_complexity_filter	
Enable low complexity filter	False
Complexity threshold	Not available.
read_mod_options	
PolyG tail trimming	
PolyG minimum length	Not available.
PolyX tail trimming	
umi_processing	
Enable unique molecular identifer	False
UMI location	Empty.
UMI length	Not available.
UMI prefix	Empty.
cutting_by_quality_options	
Cut by quality in front (5')	False
Cut by quality in tail (3')	False
Cutting window size	Not available.
Cutting mean quality	Not available.
base_correction_options	

Input Parameter	Value
Enable base correction	True
output_options	
Output HTML report	True
Output JSON report	False
Job Resource Parameters	no

2. Salmon Tool Parameters

Input Parameter	Value
Select salmon quantification mode:	reads
Select a reference transcriptome from your history or use a built-in index?	history
s_index	
Transcripts fasta file	**gencode.v41.transcripts.fa uncompressed**
Kmer length	31
Perfect Hash	False
input	
Is this library mate-paired?	paired_collection
FASTQ Paired Dataset	No dataset collection.
Specify the strandedness of the reads	A
Type of index	quasi
Discard orphan quasi	True
Validate mappings	--validateMappings
Min Score Fraction	0.65
Sets the maximum allowable MMP extension when collecting suffix array intervals to be used in chaining. This prevents MMPs from becoming too long, and potentially masking intervals that would uncover other good quasi-mappings for the read. This heuristic mimics the idea of the maximum mappable safe prefix (MMSP) in selective alignment. Setting a smaller value will potentially allow for more sensitive, but slower, mapping.	Not available.
Match Score	2
Mismatch Penalty	4
Gap Open Penalty	5
Gap Extension Penalty	3
Mimic Bowtie 2	False
Mimic Strict Bowtie 2	False
Hard Filter	False
Consensus Slack	0
Allow Dovetail	False
Recover Orphans	False
Write Mappings to Bam File	False
Consistent Hits	False
Quasi Coverage	Not available.
File containing a mapping of transcripts to genes	
Perform sequence-specific bias correction	True

Input Parameter	Value
Perform fragment GC bias correction	True
incompatPrior	0.0
Meta	False
adv	
skipQuant	False
Dump equivalence class counts	False
Dump equivalence class counts including rich weights	False
Minimum assigned fragments	Not available.
The value at which the fragment length PMF is down-sampled when evaluating GC fragment bias.	5
The maximum fragment length to consider when building the empirical distribution.	1000
The mean used in the fragment length distribution prior	250
Standard deviation	25
The forgetting factor used in the online learning schedule.	0.65
Initialization with uniform parameters	False
Maximal read mapping occurence	100
No length correction	False
Disable effective length correction	False
Ignore fragment length distribution	False
[experimental] : If this option is enabled, then no (lower) threshold will be set on how short bias correction can make effecctive lengths.	False
Number of fragment mappings to use when learning the sequence-specific bias model.	2000000
The first numAuxModelSamples are used to train the auxiliary model parameters.	5000000
The first numPreAuxModelSamples will have their assignment likelihoods and contributions to the transcript abundances computed without applying any auxiliary models.	5000
Use the traditional EM algorithm for optimization in the batch passes.	False
Range of factorization bins	0
Number of Gibbs sampling rounds to perform.	0
No gamma draw	False
Number of bootstrap samples to generate. Note: This is mutually exclusive with Gibbs sampling.	0
Bootstrap reproject	False
Thinning factor	16
The prior will be interpreted as a transcript-level prior.	False
Significant Digits	3
The prior that will be used in the VBEM algorithm.	1e-05
Write orphan links	False
Write the names of un-mapped reads to the file unmapped_names.txt.	False
Job Resource Parameters	no

Supplementary Table 5. Gene markers used for identification of macrophage subpopulations with scType

TissueType	cellName	geneSymbolmore1	geneSymbolmore2	shortName
Immune system	M2 Macrophage	CD209,ADORA3,STAT6,SOCS3,IL10,IRF4	Null	PMID: 32699181
Immune system	M1 Macrophage	CD86,IL6,PTGS2	Null	Cell marker 2.0
Immune system	Monocyte	CD14,LYZ	Null	Cell marker 2.0
Immune system	M0 Macrophage	CD68,ITGAM	Null	Cell marker 2.0

Supplementary Table 6. Information of Cases

Age	Gender	Dignosis	Grade
23	Male	Lumbar disc herniation	I-II
21	Male	Spine burst fractures	I-II
56	Female	Lumbar disc herniation	III
43	Male	Lumbar disc herniation	III
34	Male	Lumbar disc herniation	I-II
48	Male	Lumbar disc herniation	III
20	Male	Lumbar disc herniation	I-II
64	Female	Lumbar disc prolapses	IV
75	Male	Lumbar disc herniation	IV
27	Male	Lumbar disc herniation	III
37	Female	Lumbar disc herniation	IV
67	Male	Lumbar disc herniation	IV
66	Female	Lumbar disc prolapses	V
63	Female	Lumbar disc herniation	V
82	Female	Lumbar disc prolapses	V
56	Female	Lumbar disc prolapses	V

Supplementary Table 7. Intersection of ISR-Gene and DEG-Gene

No.	Intersect genes	No.	Intersect genes	No.	Intersect genes	No.	Intersect genes	No.	Intersect genes	No.	Intersect genes
1	DDIT4	59	DUSP23	117	RBFOX3	175	PRDX5	233	NOP53	291	PILRB
2	GDF15	60	DAPK3	118	NDUFB7	176	RPLP0	234	RSBN1L	292	ECHS1
3	TRIB3	61	DNPH1	119	ECH1	177	WNT5B	235	KBTBD6	293	ZFP36L2
4	MKNK2	62	JUN	120	COL15A1	178	MTSS2	236	NPIPB4	294	RARS1
5	ATF4	63	NMRAL2P	121	TTN	179	RPL35	237	COX6A1	295	C1QTNF5
6	ZC3HAV1	64	HOXC11	122	NOP58	180	RPL10	238	RPS13	296	COX8A
7	NFKBID	65	IER5L	123	RPL18	181	PNRC1	239	FLOT1	297	RINT1
8	PPP1R15A	66	LARP6	124	GLI3	182	RPS11	240	NNMT	298	C3orf38
9	SLC1A5	67	CBLN3	125	NFATC2	183	RPL3	241	DMXL2	299	FRS2
10	SLC3A2	68	SOCS3	126	RPL8	184	LENG8	242	HHLA3	300	POLE4
11	LURAP1L	69	RFXANK	127	ALKBH7	185	DNHD1	243	GPX4	301	BLOC1S1
12	GADD45B	70	GPX1	128	ECI1	186	GALM	244	HINT1	302	RPL27
13	PIM3	71	DIRAS1	129	RPS3	187	PXMP2	245	KMT2E	303	SH3BP5L
14	ACKR3	72	ERF	130	PHC2	188	RPL13A	246	EEF1G	304	ASPSCR1
15	BCL3	73	ATP6V1F	131	BDKRB1	189	FAU	247	CILK1	305	AEBP1
16	JUNB	74	VASN	132	UBA52	190	FBXL14	248	RSAD2	306	IER2
17	PLAU	75	LGR4	133	PKN1	191	FTL	249	IMPDH2	307	ASPN
18	OAS1	76	RPS16	134	UNC5B	192	WDR13	250	LBR	308	HDAC11
19	NOS2	77	PHACTR1	135	ECSIT	193	S100A6	251	ASH1L	309	RPL7A
20	MFSD3	78	SBSN	136	TNFRSF1B	194	GOLGA6L4	252	CFI	310	FSD1L
21	SPSB1	79	RESF1	137	IL6R	195	GNB2	253	MEGF10	311	GSN-AS1
22	TIMP3	80	RPLP1	138	CAPG	196	CP	254	AXL	312	DDX49
23	ID3	81	RPS29	139	PNN	197	GTPBP6	255	ASL	313	MAZ
24	ZFP36	82	UBXN1	140	TSR3	198	MEG8	256	NKTR	314	SERPINA10
25	NR4A1	83	PLGLB2	141	RAB11FIP1P1	199	CFD	257	GGH	315	HERC1
26	ZNF223	84	USE1	142	RPS2	200	SLC35F6	258	HOXA-AS3	316	USP24
27	PDZRN3	85	NAV3	143	PYGB	201	DDX46	259	RPS20	317	SLC25A1P5
28	PPP4R4	86	EIF1	144	HOTAIR	202	KRT14	260	EEF1A1P6	318	DGCR6L
29	STK40	87	RNF19B	145	BCKDHA	203	TJP2	261	SLCO4C1	319	MIR4280HG
30	TNFSF15	88	PGLS	146	LGALS1	204	ITPK1	262	COX7A2	320	NOD2
31	ITPKC	89	MAT2A	147	MTCO1P12	205	RPL37A	263	RPSA	321	FZR1
32	SARS1	90	RPL18A	148	RPS14	206	MRPL54	264	RPL36	322	THBD
33	CEBPB	91	CDC42EP1	149	VPS51	207	SNTA1	265	NME2	323	EPHX1

34	GRIP2	92	CHASERR	150	MMP1	208	BSG	266	LINC00665	324	SQSTM1
35	NPM3	93	RPS28	151	DENND4C	209	VAT1	267	FBL	325	SAE1
36	GPR78	94	EFNA1	152	ZNRD1ASP	210	HSD17B14	268	LINC00963	326	RPL29
37	H1-10	95	BTG2	153	CD274	211	ASAP1	269	TRIM16L	327	LINC01018
38	H19	96	MAFG	154	SLC25A6	212	DCBLD2	270	CORO2B	328	MCM7
39	STK24	97	JUND	155	MRPL34	213	ZNF266	271	SMARCA1	329	MRPL47
40	HAS2	98	RPS19	156	PTGES2	214	RPL28	272	RRP9	330	RPS17
41	TNFSF10	99	EVA1B	157	STOML2	215	LINC02371	273	PTMS	331	ZNF326
42	RGS17	100	SNX15	158	CAVIN3	216	TCEAL9	274	CHCHD2	332	KRT7
43	HMOX1	101	CTSS	159	EIPR1	217	MRPL39	275	GPRC5C	333	PPP1R14B-AS1
44	TRIM38	102	RPS9	160	EEF1D	218	C12orf57	276	PTGFR	334	LPAR4
45	DTX3L	103	SNHG28	161	RPS15	219	DLEU2	277	ARF5	335	DDAH2
46	BHLHE40	104	SNHG32	162	LINC02582	220	POLR2I	278	PTPN3	336	PFN1
47	LINC02605	105	HIPK2	163	MRPL53	221	OAZ1	279	SESN3	337	GAPDH
48	HTR2A	106	TIA1	164	RPL13	222	RPS21	280	GAS5	338	ARL6IP4
49	ID1	107	EEF2	165	PLP2	223	FLNC	281	RAPH1	339	TP53
50	KRT86	108	TNFRSF12A	166	AGO4	224	WDR83OS	282	ADCY4	340	HSP90AA1
51	MYC	109	RNH1	167	CITED4	225	RPLP2	283	UXT	341	PHB2
52	BLVRB	110	CMTR2	168	MRPL24	226	IRF2BPL	284	AIFM3	342	CASP9
53	KLF10	111	RPS10	169	SEPHS2	227	RACK1	285	KLHL21	343	CRYZ
54	LAGE3	112	MIR100HG	170	HOMER3	228	MIF	286	SSR4	344	ESYT1
55	CXCL3	113	RPS5	171	DDI2	229	UBR2	287	FIS1	345	MDGA1
56	PARP14	114	PHIP	172	MDM2	230	KRT8	288	CACNB2	346	ZNF23
57	NFKBIZ	115	ROMO1	173	FOSL2	231	DGKH	289	SEMA7A	347	EMC10
58	C8orf82	116	FEZF1-AS1	174	EIF3C	232	ANGPT1	290	SNHG29	348	SRP54

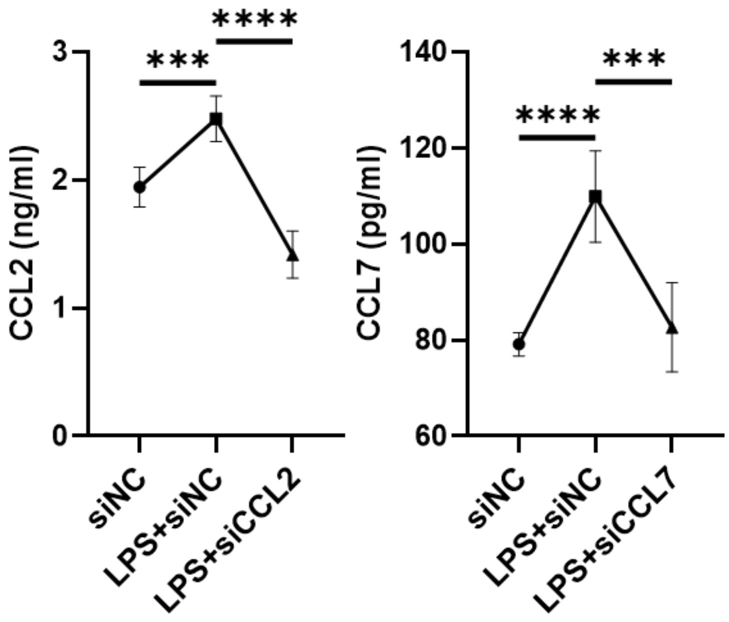

Supplementary Fig. 1 Effect of CCL2 and CCL7 siRNA on CCL2&7 protein levels in rNPCs versus negative control siRNA. Experiments were performed 3 times and data are presented as means ± SD. *, p < 0.05; **, p < 0.01; ***, p < 0.001; ****, p < 0.0001; ns:not significant, Two-way ANOVA.

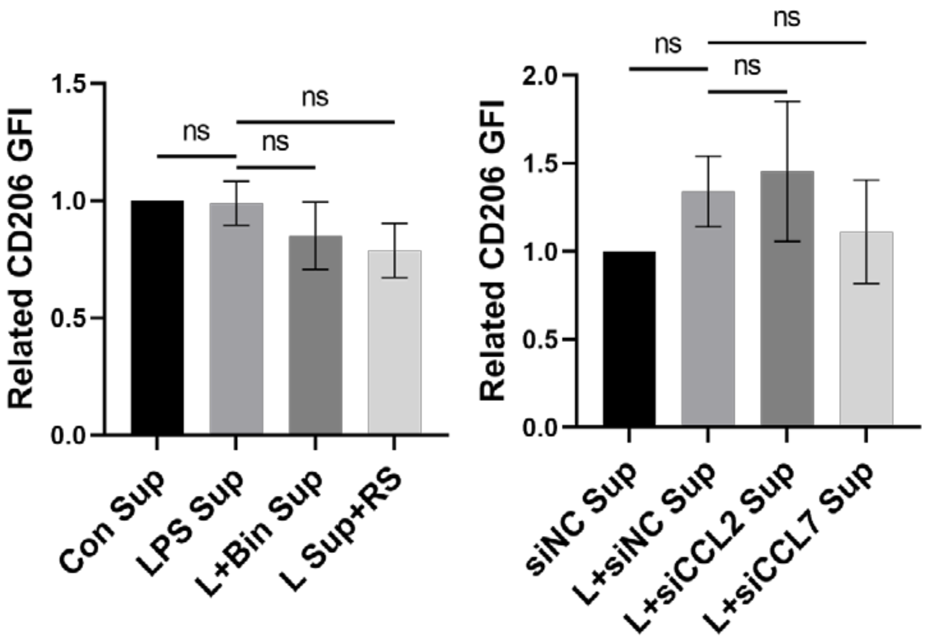

Supplementary Fig. 2 Histogram represent GFI for CD206 proteins in rMΦs pretreated with supernatant from rNPCs pretreated with siRNAs, Bindarit or RS102895 and LPS. Experiments were performed 3 times and data are presented as means ± SD. ns, not significant, with ANOVA.

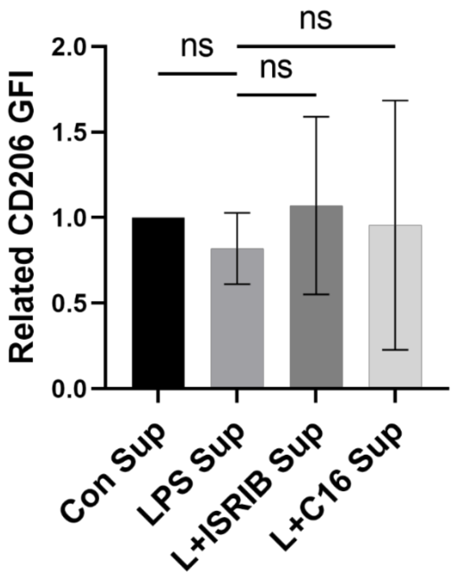

Supplementary Fig. 3 Histogram represent GFI for CD206 proteins in MΦs treated with supernatant from NPCs treated with ISRIB or C16 and LPS. Experiments were performed 3 times and data are presented as means ± SD. ns, not significant, with ANOVA.

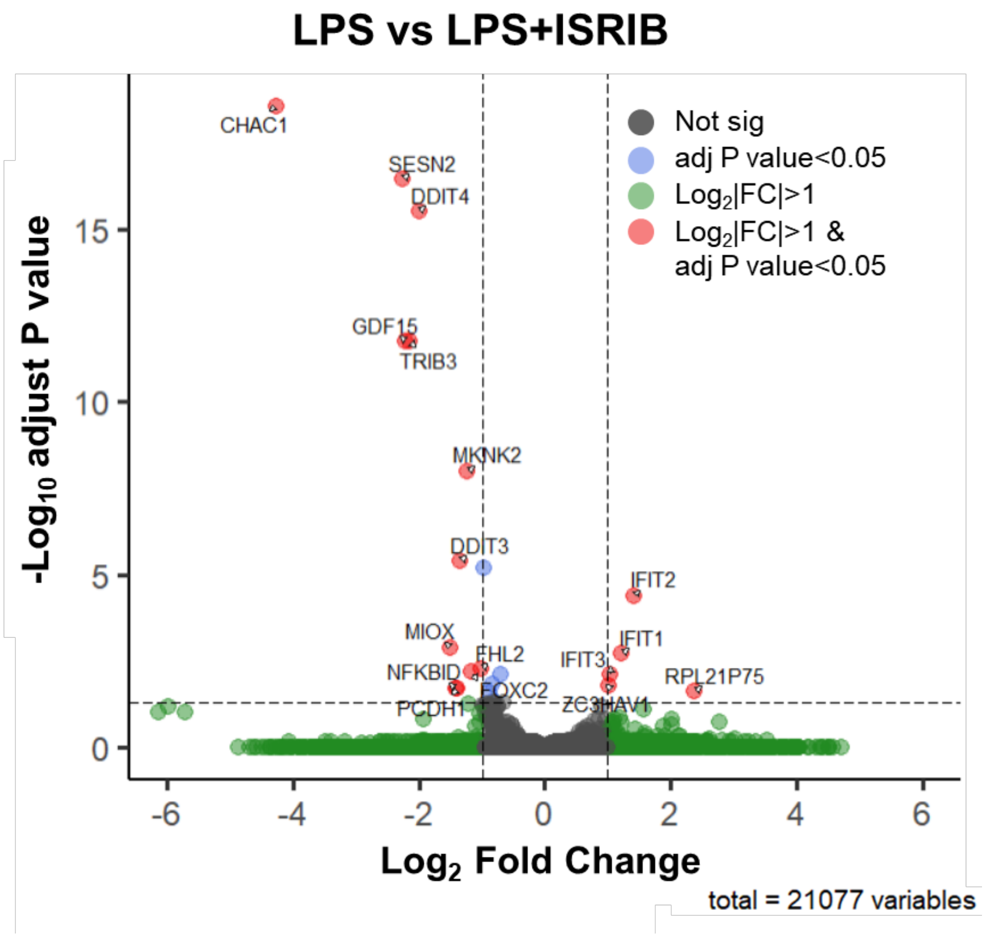

Supplementary Fig. 4 The volcano diagram shows the differential expression analysis results of transcriptome of NPCs with or without ISRIB pretreatment and LPS treatment

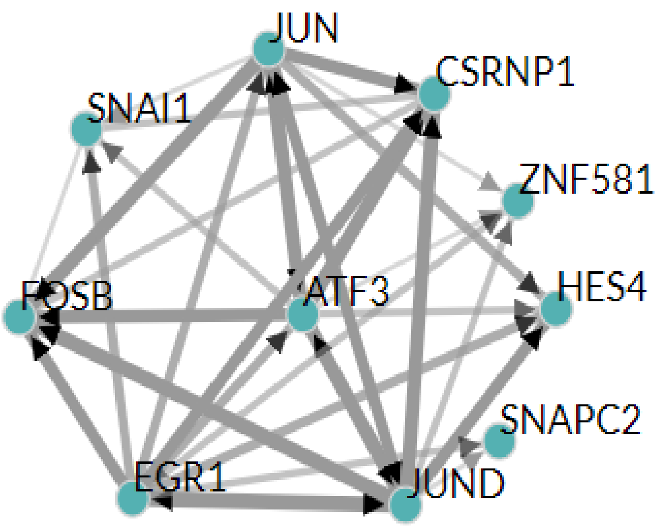

Supplementary Fig. 5 Correlation analysis of transcription factors in CheA3

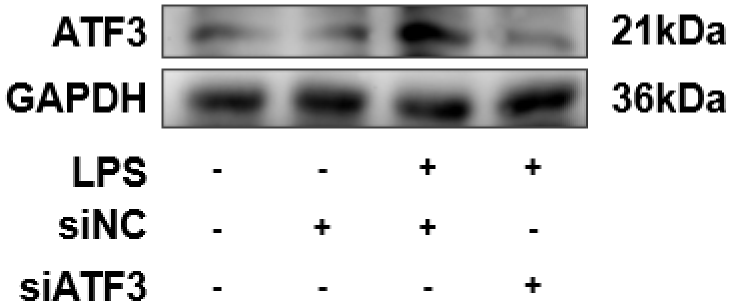

Supplementary Fig. 6 WB result showed effect of ATF3 siRNA on ATF3 protein levels in NPCs versus negative control siRNA.

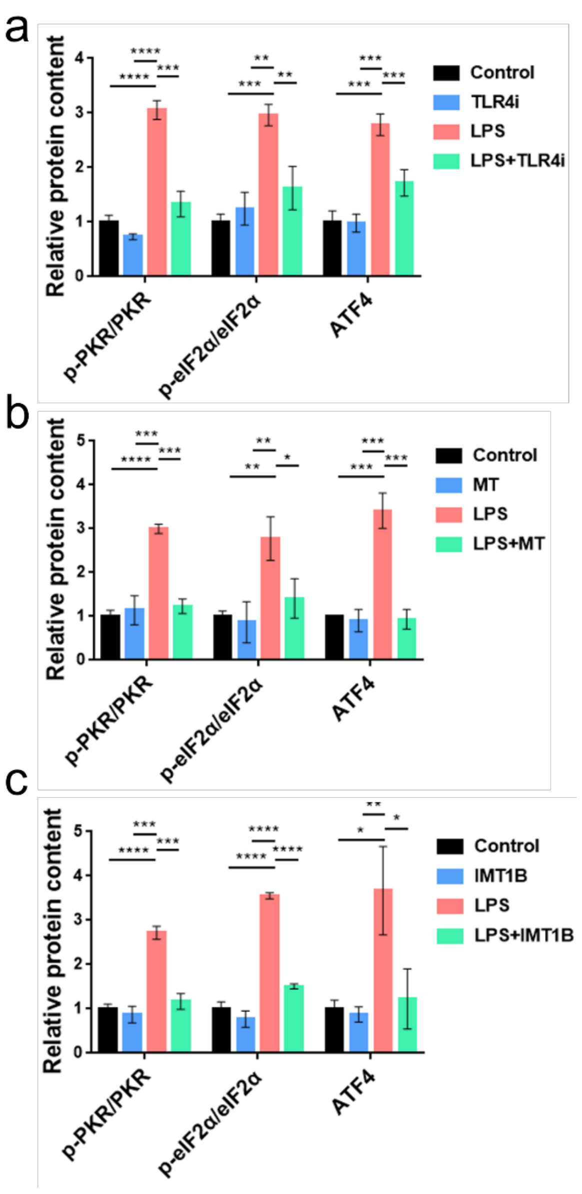

Supplementary Fig. 7 The activation of ISR is modified by TLR4-mROS-dsRNA axis. The quantitative analysis of WB of ISR-related proteins in response to the stimulation of TLR4-IN-C34 (TLR4i, **a**), mitoTempo (MT, **b**), and IMT1B (**c**). Experiments were performed 3 times independently, and data are presented as means ± SD. *, $p < 0.05$; **, $p < 0.01$; ***, $p < 0.001$, ****, $p < 0.0001$; ns, not significant, with ANOVA.

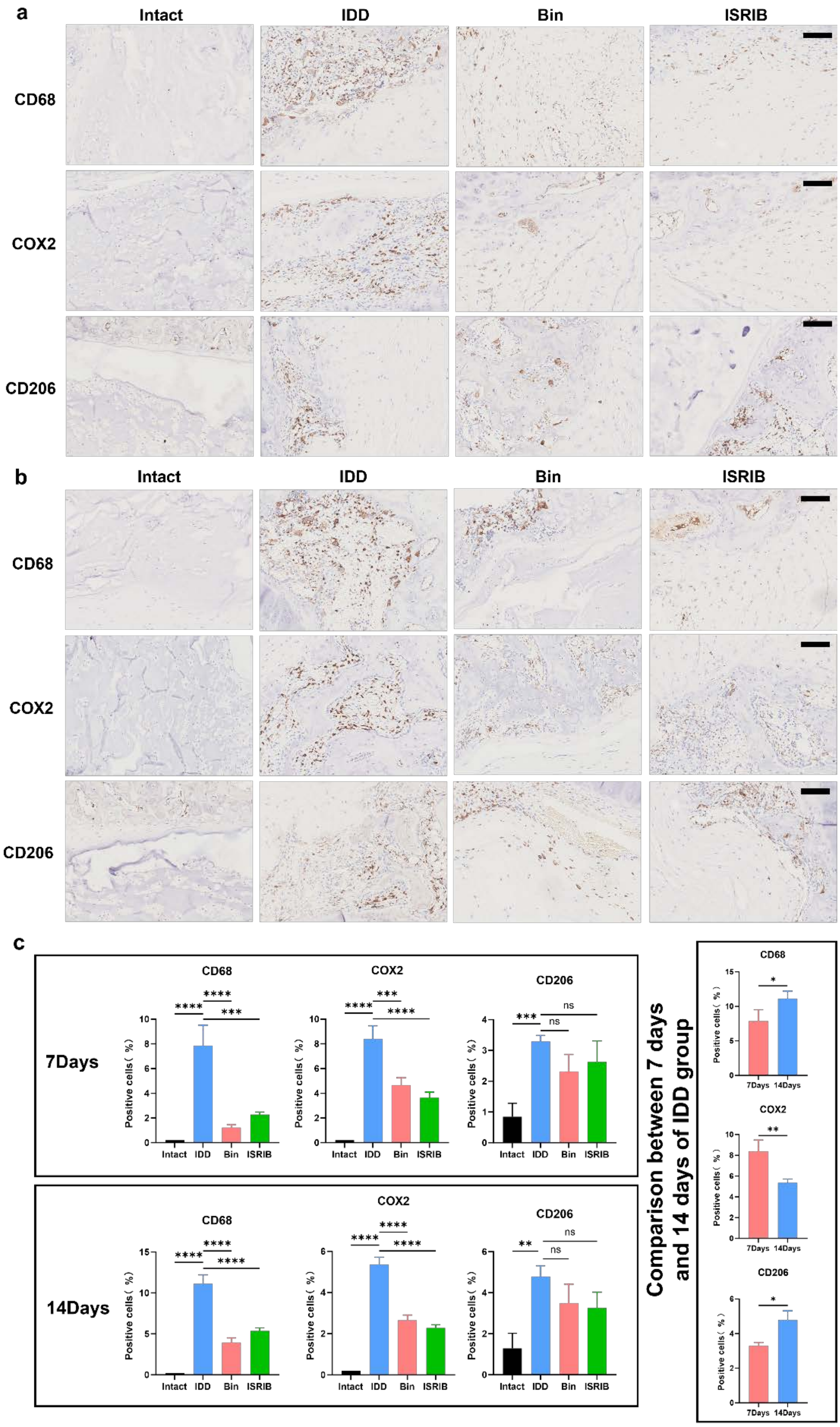

Supplementary Fig. 8 Representative immunohistochemical images and quantification analysis of CD68, COX2 and CD206 in each treatment group of rat IVD tissues in early stage.

a Representative IHC results of CD68, COX2 and CD206 in each treatment group of rat IVD tissues on Day 7 after puncture. **b** Representative IHC results of CD68, COX2 and CD206 in each treatment group of rat IVD tissues on Day 14 after puncture. **c** Qualification of CD68,CD206 and COX2 in IHC experiments. Experiments were performed 3 times independently and data are presented as means ± SD. *:p < 0.05; **:p < 0.01; ***:p < 0.001; ns: not significant, with ANOVA or T-test.

Other materials and methods

ELISA

The levels of CCL2 and CCL7 in the supernatant of NPCs were detected by CCL2/7 ELISA kits (Ruixin Biotech, China). All processes followed the manufacturer's standard operating procedures. The absorbance was detected by an F50 enzyme labeling instrument (TECAN, Switzerland) and converted into content by the standard curve method.

Protein extraction and western blotting (WB) detection

A mixture of proteolysis solution and protease inhibitor (NCM Biotech, China) was used to collect the total cellular protein. Then, the protein concentration was determined by a BCA kit (Biosharp, China). Then, protein samples were mixed with 5× loading buffer (Solarbio, China) and denatured by heating (90 °C, 10 min). Next, the denatured samples were loaded into 10% or 15% SDS-PAGE gels (Epizym, China), transferred to methanol-activated PVDF membranes (Millipore, USA) and blocked at 25 °C with 5% BSA for 1 h. The membranes were then immunoblotted with primary antibodies, including rabbit-anti-ATF4 (1:1000, A18687, Abclonal, China), rabbit-anti-eIF2α (1:1000, A0764, Abclonal, China), rabbit-anti-phospho-eIF2α-S51 (1:1000, A0692, Abclonal, China), mouse-anti-GAPDH (1:5000, AC002, Abclonal, China), rabbit-anti-PERK (1:1000, A18196, Abclonal, China); rabbit-anti-GCN2 (1:1000, A7155, Abclonal, China), rabbit-anti-HRI (1:500, A14119, Abclonal, China), rabbit-anti-PKR (1:1000, ab184257, Abcam, USA); rabbit-anti-phospho-PKR-T446 (1:500, bs-3335R, Bioss, China), or rabbit-anti-ATF3 (1:1000, A13469, Abclonal, China) overnight at 4°C. The membranes were then washed with TBST and incubated with secondary antibody (HRP goat-anti-rabbit IgG, 1:5000, AS014, Abclonal, China; HRP goat-anti-mouse IgG, 1:5000, PR30009, Proteintech, USA) at 25 °C for 1 h. The bands were detected using a ChemDoc imaging system (Bio-Rad, USA).

Flow cytometry (FCM) analysis

The treated MΦs were digested with 0.25% trypsin (Gibco, USA) and transferred to a flow tube (BD Biosciences, USA) at 2*10^4 cells/tube. All operations were performed in accordance with the standard protocol provided by BD Biosciences. For rat MΦs, an anti-rat CD16/32 antibody (550270, BD Biosciences, USA) was used to block any nonspecific FcR binding. FITC anti-rat CD11b/c antibody (201805, Biolengend, USA) and APC anti-rat CD68 antibody (sc20060, Santa Cruz, USA) were used to identify MΦ clusters. BV421 anti-rat CD86 (743211, BD Biosciences, USA) and PE anti-rat CD206 (sc58986, Santa Cruz, USA) were used to distinguish M1 and M2 MΦs. For mouse MΦs, we used anti-mouse CD16/32 (101319, Biolegend, USA) to block nonspecific FcR binding. APC anti-mouse F4/80 (123115, Biolegend, USA) was used to identify MΦ clusters. PerCP anti-mouse CD86 (105027, Biolegend, USA) and PE anti-mouse CD206 (141705, Biolegend, USA) were used to distinguish M1 and M2 MΦs. All tubes were detected by CytoFlex flow cytometry (Beckman, USA). All data were analyzed and quantified by Flowjo software (https://www.flowjo.com/).

Hematoxylin and eosin (HE) and Safranin O-Fast Green (SO) staining

For HE staining, dewaxed paraffin sections were stained with hematoxylin solution and then differentiated with 8% hydrochloric acid-alcohol differentiation solution at 25 °C for 2 s. The sections were then treated with bluing buffer for 4 min and washed in 95% alcohol. Next, the sections were soaked in eosin dye for 2 s and washed with anhydrous alcohol. For SO staining, the dewaxed paraffin sections were stained with fresh Weigert solution for 5 mins and then differentiated by 8% hydrochloric acid-alcohol differentiation solution at 25℃ for 15 s. The sections were then stained for 5 mins with fast green solution and washed with pure water until the cartilage tissue was colorless. Next, safranin O solution was used to stain until the section cartilage was bright red. Finally, the stained sections were blocked with neutral gum and visualized by an Olympus BX51 microscope (Olympus, Japan).

Immunohistochemical (IHC) staining of paraffin sections

After the paraffin sections were dewaxed, the antigen was repaired with pH 9.0 EDTA for 2 min. Paraffin sections were then treated with 3% H_2O_2 for 20 min to block endogenous peroxidase at room temperature. After washing with TBS, sections were blocked for 1 h with 10% goat serum (Boster, China), followed by incubation in primary antibody working solution at 4 °C overnight. The primary antibodies used were as follows: rabbit-anti-phospho-eIF2α-S51 1:200 (AP0745, Abclonal, China), rabbit-anti-COX2 (1:200, A1253,

Abclonal, China), mouse-anti-CD68 (1:200, ab201340, Abcam, USA), rabbit-anti-CD68 (1:200, BA3638, Boster, China), rabbit-anti-CD86 (1:200, 19589, CST, USA), and rabbit-anti-CD206 (1:200, A8301, Abclonal, China). The secondary antibody DAKO K5007 (DAKO, Denmark) was added to sections after rewarming and rinsing with TBS. After incubating for 25 min at 25 °C, the sections were washed with TBS and stained with 50 µl of DAB (DAKO, Denmark). Then, the sections were rinsed with water, stained with hematoxylin, rinsed with water and differentiated in 1% hydrochloric acid alcohol for 2 s. The sections were then treated with bluing buffer (Bios Biological, China), dried by alcohol and decolorized with xylene for 30 min. Finally, the sections were blocked with neutral gum and imaged by an Olympus BX51 microscope (Olympus, Japan). ImageJ 1.53 (https://imagej.net/Fiji) was used for quantitative analysis of the percentage of positively stained cells.

Immunofluorescence (IF) analysis

Cells were washed with PBS and fixed with 4% paraformaldehyde for 15 min. Fixed cells were permeabilized with 0.5% Triton X-100 in PBS for 10 min. Then, the cells were blocked with goat serum (Boster, China) at 25 °C for 1 h and incubated with primary antibody working solution [J2 antibody (1:250, 10010200, Nordic Mubio, Holland); rabbit-anti-TOM20 antibody (1:200, A19403, Abclonal, China)] for 2 h. Then, the cells were washed with PBST and incubated with secondary antibody working solution [AF488 goat-anti-rabbit IgG antibody (1:300, AS053, Abclonal, China), AF594 goat-anti-mouse IgG antibody (1:300, AS054, Abclonal, China)] at 25 °C for 1 h. Finally, the cells were blocked with anti-quenching medium (Boster, China) after being dyed with DAPI solution (Beyotime, China) for 15 min. A BX51 fluorescence microscope and CellSens software (Olympus, Japan) were used for imaging and to analyze the images, respectively.

For IF analysis of tissue samples, after dewaxing and dehydration, the slides were incubated with primary antibodies against mouse-anti-CD68 (1:200, ab201340, Abcam, USA), rabbit-anti-CD68 (1:200, BA3638, Boster, USA), rabbit-anti-COX2 (1:200, A1253, ABclonal, China), mouse-anti-COX2 (1:200, sc-376861, Santa Cruz, USA), rabbit-anti-F4/80 (1:200, 70076, CST, USA), goat-anti-CD206 (1:200, AF2535, R&D, USA), and mouse-anti-CCR2 (1:200, sc-74490, Santa Cruz, USA). Fluorescence-conjugated secondary antibodies (Invitrogen, USA) were used to detect fluorescent signals before counterstaining with DAPI (Beyotime, China). An anti-fluorescence quenching sealer (Thermo Fisher Scientific) was dropped onto the slides to prevent fluorescence quenching. Sample fluorescence signals were captured using a fluorescence microscope (Olympus, Tokyo, Japan).
